# The chromosome-level genomes of two *Macaranga* species provide insights into the molecular mechanism of nervonic acid accumulation

**DOI:** 10.1093/hr/uhag114

**Published:** 2026-04-02

**Authors:** Dong-Hai Li, Qi Qiang, Zheng-Shan He, Fa-Yi Chen, Bao-Zheng Chen, Ya-Min Xu, Juan Guo, Jun-Bo Yang, Wen-Bin Yu, Yao-Huan Xu, Yu-Jiao Wang, Li Yang, De-Zhu Li, Bo Tian

**Affiliations:** Laboratory of Tropical Plant Resources and Sustainable Use, Xishuangbanna Tropical Botanical Garden, Chinese Academy of Sciences, Mengla, Yunnan 666303, China; Laboratory of Tropical Plant Resources and Sustainable Use, Xishuangbanna Tropical Botanical Garden, Chinese Academy of Sciences, Mengla, Yunnan 666303, China; Germplasm Bank of Wild Species & Yunnan Key Laboratory of Crop Wild Relatives Omics, Kunming Institute of Botany, Chinese Academy of Sciences, Kunming, Yunnan 650201, China; Department of Bioinformatics Analysis, Wuhan Generead Biotechnologies Co. Ltd., Wuhan Software New City Phase 3, Huacheng Avenue, Hongshan District, Wuhan, Hubei 430076, China; College of Food Science and Technology, Yunnan Agricultural University, Kunming, Yunnan 650201, China; State Key Laboratory of Phytochemistry and Natural Medicines, Kunming Institute of Botany, Chinese Academy of Sciences, Kunming, Yunnan 650201, China; Laboratory of Tropical Plant Resources and Sustainable Use, Xishuangbanna Tropical Botanical Garden, Chinese Academy of Sciences, Mengla, Yunnan 666303, China; Laboratory of Tropical Plant Resources and Sustainable Use, Xishuangbanna Tropical Botanical Garden, Chinese Academy of Sciences, Mengla, Yunnan 666303, China; Germplasm Bank of Wild Species & Yunnan Key Laboratory of Crop Wild Relatives Omics, Kunming Institute of Botany, Chinese Academy of Sciences, Kunming, Yunnan 650201, China; Center for Integrative Conservation and Yunnan Key Laboratory for the Conservation of Tropical Rainforests and Asian Elephants, Xishuangbanna Tropical Botanical Garden, Chinese Academy of Sciences, Mengla, Yunnan 666303, China; Department of Bioinformatics Analysis, Wuhan Generead Biotechnologies Co. Ltd., Wuhan Software New City Phase 3, Huacheng Avenue, Hongshan District, Wuhan, Hubei 430076, China; Laboratory of Tropical Plant Resources and Sustainable Use, Xishuangbanna Tropical Botanical Garden, Chinese Academy of Sciences, Mengla, Yunnan 666303, China; Laboratory of Tropical Plant Resources and Sustainable Use, Xishuangbanna Tropical Botanical Garden, Chinese Academy of Sciences, Mengla, Yunnan 666303, China; Germplasm Bank of Wild Species & Yunnan Key Laboratory of Crop Wild Relatives Omics, Kunming Institute of Botany, Chinese Academy of Sciences, Kunming, Yunnan 650201, China; Center for Interdisciplinary Biodiversity Research & College of Forestry, Shandong Agricultural University, Tai’an, Shandong 271018, China; Laboratory of Tropical Plant Resources and Sustainable Use, Xishuangbanna Tropical Botanical Garden, Chinese Academy of Sciences, Mengla, Yunnan 666303, China

## Abstract

Nervonic acid (NA) is a monounsaturated fatty acid (FA) with multiple therapeutic and hygienic benefits, and accumulates in plant seeds. It is essential for developing innovative plant sources of NA to elucidate the molecular regulation mechanisms underlying NA biosynthesis and accumulation. We present high-quality chromosome-level *de novo* genome assemblies of two contrasting NA-producing plants: high-NA *Macaranga indica* and low-NA *M. denticulata*. Both *M. indica* and *M. denticulata* retained considerable repetitive sequences, similar chromosome arrangements, and analogous regulation models in capsule maturation. Correlated to faster accumulation of NA, *M. indica* concentrated genes and regulators of FA biosynthesis, FA elongation and triacylglycerol biosynthesis in three ways: uniform regulators, fewer analogous duplications and higher expression of genes. In contrast, *M. denticulata* showed a diverse environment response, and had more analogous redundant duplications. Despite their sympatric distribution, the two species showed staggered flowering. Cold, ABA and phosphate starvation were major elicitors in *M. indica*, and core genes were identified as *KCS*, *PDAT*, and *DGAT*. Further resequencing analysis of *M. indica* revealed geography-specific gene polymorphisms and migration pathways. Our results unveil the differences in NA biosynthesis and accumulation mechanisms between *M. indica* and *M. denticulata*. This understanding, combined with insights into cultivation strategies and regional genetic diversity, provides a basis for the domestication and breeding of *Macaranga* species toward efficient NA production.

## Introduction

Nervonic acid (NA, 15*Z*-tetracosenoic acid), a monounsaturated very long-chain fatty acid (VLCFA), serves as a critical component of nerve fibers during brain development and demonstrates therapeutic potential in alleviating and counteracting several neurodegenerative diseases [1–3]. Neural proliferation in the adult brain creates demand for NA [4]. However, postnatal humans cannot synthesize NA endogenously and therefore require exogenous supplementation [2, 5]. Over recent decades, NA shortage has been exacerbated by bans on fishing for sharks, a primary animal source of NA, coupled with inefficient chemical synthesis yielding excessive by-products [6]. Although plant-derived NA mitigates the current predicaments of supply constraint, a few NA-producing species have been introduced into horticulture but have not been domesticated in the traditional agricultural sense, giving low yields with miscellaneous obstructions from scarce and restricted habitats and long juvenile phases (e.g. *Malania oleifera* [7]); vulnerable seeds and obligate requirement for red soil (e.g. *Cardamine graeca* [8]); difficult sowing and propagation (e.g. *Tropaeolum speciosum* [8]); or low yield with non-negligible erucic acid content (e.g. *Lunaria annua* [9]). Consequently, systematic investigations of alternative plant species with improved cultivation practices are required to advance both industrial-scale NA biosynthesis and mechanistic understanding of NA accumulation. In terms of genetic studies, although fragmentary molecular mechanisms of NA biosynthesis have been identified in certain plants, the core genes, their regulation, and influencing factors remain obscure [6].

Previous research revealed that substantial variations in NA content exist in seed oils of *Macaranga* species, including *M. indica* and *M. denticulata* [10]. The paleotropical genus *Macaranga* Thouars (Euphorbiaceae) comprises over 300 species and ranges from Africa across Southeast Asia to the Pacific islands [11]. Most *Macaranga* species inhabit rainforest edges or the peripheries of human-modified sites. They exhibit key pioneer traits, such as being light-demanding and fast-growing [12–14]. Both *M. indica* and *M. denticulata* are widely distributed across South and Southeast Asia, while *M. denticulata* extends to South India and Sri Lanka and *M. indica* ranges from the Himalaya to the Indonesian Archipelagos (Supplementary Fig. S1F) [15]. They are phylogenetically closely related [16,17]. As a pioneer species [18], *M. indica* possesses exceptional resource potential owing to its broad distribution, high genetic diversity, elevated fecundity, precocious maturity, and advantageous seed traits. Notably, seed oil of *M. indica* contains over 30% NA [10]. In contrast, the sibling species *M. denticulata* exhibited a low NA content of merely 6.5%, yet its total oil content was comparable [19].

Therefore*,* the divergent NA contents but ecologically similar phenotypes of *M. indica* and *M. denticulata* provide an exceptional comparative system for identifying NA biosynthesis genes while filtering background genetic noise. In response to biotic and abiotic stress, *Macaranga* species evolved discrete adaptation strategies for their respective environments [20]. Lipids are essential for plants to accommodate environments and interact with peripheral microorganisms [21, 22]. Their composition has characteristic features to acclimate the plants to different circumstances, and, critically, lipid synthesis/metabolism and stress tolerances are closely associated as well. Therefore, it is critical to decipher the genomes of the two species to elucidate their differential metabolic potentials via comparative genomic analysis.

From the perspective of NA biosynthesis, like other VLCFAs, NA biosynthesis involves two main processes: *de novo* fatty acid (FA) biosynthesis and FA elongation. FA elongation determines the specificity of VLCFAs and its catalysis mainly occurs in the endoplasmic reticulum (ER): long-chain acyl-coenzyme A synthetases (LACSs) activate and generate malonyl-CoA (C16–C18) [23]; FA elongation from C16/C18 is catalysed by the FA elongation complex (FAE), consisting of β-ketoacyl-CoA synthase (KCS), β-ketoacyl-CoA reductase (KCR), β-hydroxyacyl-CoA dehydratase (HCD), and enoyl-CoA reductase (ECR) [24]. KCSs catalyse the incipient, rate-limiting step in the FA elongation loop, thereby determining substrate specificity and final VLCFA product profiles [25, 26]. Following sequential condensation (KCS), reduction (KCR), dehydration (HCD), and a second reduction (ECR) [27], the FA-CoA substrates are elongated by two carbons on its FA moiety. Then the free FA-CoAs are transferred to a glycerol backbone [28], and ultimately form TAG, deposited in lipid droplets [29], or are transformed to phospholipid, sphingolipid, galactolipids, cutin, suberin, and waxes [30]. Within the FA elongation pathway, rather than KCR, HCD, or ECR [31], KCSs exhibit significant heterogeneity in cellular/tissue localizations, substrates/products and stress/development regulation [32–34]. Current mechanistic studies on NA biosynthesis primarily focus on *Camelina sativa* [35], *Cardamine graeca* [8]*, L. annua* [9], and *M. oleifera* [7], However, the fundamental KCSs responsible for NA synthesis in *Macaranga* species, whether FAE/KCS18 or KCS11, remain ambiguous. Furthermore, the regulatory mechanisms governing these KCS enzymes are yet uncharacterized.

Triacylglycerol, the primary storage form of FA in seeds, consists of a glycerol backbone esterified with three FA moieties by acyltransferase [36]. In the process of TAG biosynthesis in plants, diacylglycerol (DAG) is transferred to TAG by diacylglycerol *O*-acyltransferase (DGAT) in the Kennedy pathway [37], or by phospholipid:diacylglycerol acyltransferase (PDAT) [38], assembling one acyl group to the carbon glycerol backbone at the sn-3 position. This transformation step is rate-limiting for TAG biosynthesis with substrate diversity [39]. PDAT catalyses TAG formation via an acyl-CoA-independent pathway, specifically transferring acyl groups from the sn-2 position of phospholipids (primarily phosphatidylcholine) to DAG, yielding an sn-1-lysophospholipid and a TAG molecule [40]. As an isoenzyme of PDAT, DGAT performs the terminal and sole committed step in TAG biosynthesis, utilizing DAG and acyl-CoA as substrates [41, 42]. Beyond its fundamental role as an energy reserve, stored TAG significantly influences critical plant processes, including cell division/expansion, stomatal opening, membrane remodeling, reproductive development, and pollination [36]. Furthermore, TAG accumulation enhances plant tolerance and responses to diverse environmental stress, such as prolonged darkness [43], nitrogen deprivation [44], and drought [45] and heat stress [46]. However, how these environmental factors influence and are selective for specific species remains poorly understood.

Here we generated high-quality *de novo* genome assemblies for *M. indica* and *M. denticulata* using single-molecule real-time sequencing (SMRT) combined with Hi-C scaffolding and anchored these assemblies to 11 chromosomes. We employed comparative genomics and integrative multiomics analysis to identify and characterize the transcriptional and metabolic pathways driving stress response, followed by further bioinformatics exploration to dissect the differences in lipid biosynthesis between *M. indica* and *M. denticulata*. Our study provides unprecedented insights into the molecular mechanism and stimulants of plant NA biosynthesis, and would serve as a foundation for precision engineering of NA production in *M. indica* plantations.

## Results

### Genome assembly and functional annotation

**Figure 1:**
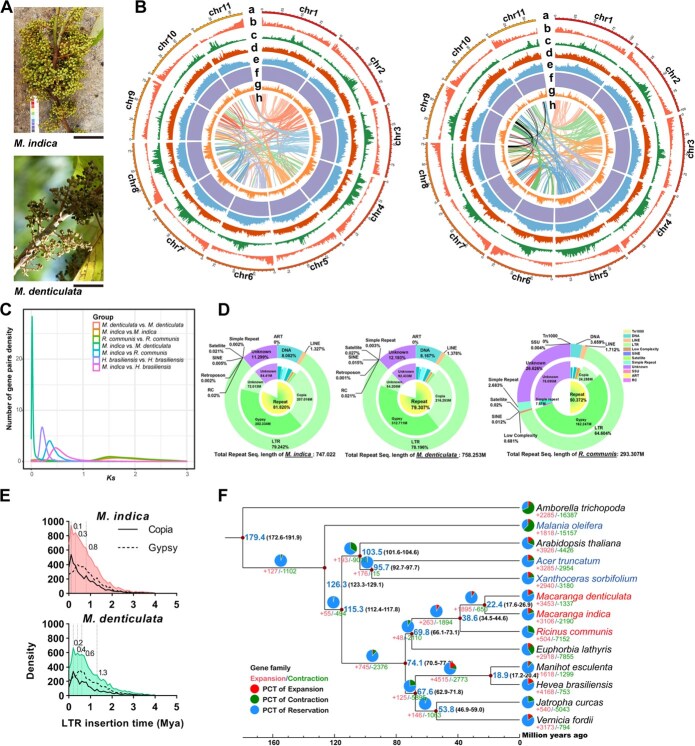
Genomic characteristics, evolution, and structural features of *M. indica* and *M. denticulata*. (A) Images of *M. indica* and *M. denticulata*. Scale bar = 10 cm. (B) Genome features of *M. indica* and *M. denticulata*. Sequence features are detailed inside-out as (a) pseudochromosomes; (b) gene density; (c) Copia-type LTR retrotransposon density; (d) Gypsy-type LTR retrotransposon density; (e) repetitive sequence density; (f) non-coding RNA density (including rRNA, snRNA, tRNA, and miRNA); (g) GC density; and the central chord diagram (h) illustrating syntenic relationships of homologous genes. (C) Ridgeline curves showing *K*_s_ distribution of paralogs in *Macaranga* and other two species of Euphorbiaceae: *M. indica*, *M. denticulata*, *Ricinus communis*, and *Hevea brasiliensis*. (D) Repetitive element composition. Innermost pie: total repetitive content; middle ring: classified elements; outer ring: subcategories. (E) Waterfall graph showing LTR retrotransposon accumulation over evolutionary time. (F) Composite dendrogram of 13 angiosperm species showing gene family expansion/contraction. Non-*Macaranga* and NA-producing species (blue text), *Macaranga* and *R. communis* (red text). Pie charts: proportions of expanded (red), contracted (green), and conserved gene families. Nodes show estimated divergence times (Mya).

*De novo* genome assembly of *M. indica* and *M. denticulata* (Fig. 1A and B) produced 41.41 Gb (*M. indica*, 45.06×) and 43.45 Gb (*M. denticulata*, 49.30×) of high-quality paired-end short reads by Illumina Hiseq X Ten, and 158.61 Gb (*M. indica*, 172.58×) and 190.38 Gb (*M. denticulata*, 216.03×) long reads by PacBio Sequel II, respectively. Hi-C libraries generated 101.23 Gb (*M. indica*, 110.14×) and 106.71 Gb (*M. denticulata*, 121.09×). The raw data of *M. indica* and *M. denticulata* with Q20 were 96.90% and 97.18% (Supplementary Tables S1 and S2). The genomes of *M. indica* and *M. denticulata* were reorganized at 986.84 Mb (40× depth) and 946.23 Mb (54× depth) in preliminary assembly and eventually at 912.80 and 955.90 Mb (Supplementary Table S3). Genomes of *M. indica* and *M. denticulata* contained 31 scaffolds; the longest contig exceeded 37.74 and 38.12 Mb, respectively, and N50 contigs were 9.75 and 9.08 Mb, respectively (Supplementary Table S3), and complete BUSCOs (Benchmarking Universal Single-Copy Orthologs) of *M. indica* and *M. denticulata* were 1324 (96.30%) and 1339 (97.30%), respectively. These results illustrated that the contigs and scaffolds were of high quality. For an overview of *M. indica* and *M. denticulata* genomes, sequences were assembled on chromosomes and the karyotype was confirmed as 2*n* = 22 by optical microscopy (Supplementary Fig. S1A). Genomic sequences of *M. indica* (98.78%) and *M. denticulata* (95.37%) by Hi-C were assigned to chromosomes (Fig. 1B; Supplementary Table S4).

Compared with the NCBI taxonomy database, *Ricinus communis* was resolved closest to *M. indica* and *M. denticulata* among the selected angiosperms (Supplementary Table S1). Using five reference databases, 22 293 genes (87.56%) of *M. indica* and 24 367 genes (88.51%) of *M. denticulata* commonly aligned (Supplementary Fig. S1B; Supplementary Table S5) with *Arabidopsis thaliana*, *Sesamum indicum* and six spurge species: *Vernicia fordii*, *Euphorbia lathyris*, *Jatropha curcas*, *Hevea brasiliensis*, *Manihot esculenta* and *R. communis*. Values of all genomic features except mRNA average length in *M. indica* were similar or smaller to those in *M. denticulata*, including mean exon length, total intron length, and total exon length (Supplementary Table S6) while they were discrepant in number of tRNAs (484 and 687), rRNAs (281 and 1237), and snRNAs (1768 and 1879) (Fig. 1B, Supplementary Table S7). Notably, *M. indica* incorporated fewer tRNAs and rRNAs, especially 18S and 5S, suggesting fewer ancestral gene duplication events occurred.

### Selective repetitive sequence-driven genomic expansion with lineage-specific genes

Our previous 17 *K*-mer analysis [47] had predicted a sizable repetitive sequence in *M. indica* and *M. denticulata* (Supplementary Table S1). The *K*_s_ distribution of paralogs (Fig. 1C) also reflected relatively active replication events between *M. indica* and *M. denticulata*, as the *K*_s_ density for *M. indica* vs *M. denticulata* was significantly higher than for other pairs. Using a combination of Tandem Repeats Finder (TRF), RepeatMasker, protein masking, and *de novo* assembly, *M. denticulata* contained more unique genes than *M. indica* (Supplementary Fig. S1C, Supplementary Table S8). We identified repetitive sequences constituting 81.82% of the *M. indica* genome and 79.31% of the *M. denticulata* genome (Fig. 1D, Supplementary Table S9). These repeat proportions were notably higher in spurge plants compared with *H. brasiliensis* (71.18%) [48], *R. communis* NSL4377 (53.35%) [49], *R. communis* WT05 (52.59%) [50], *V. fordii* (58.74%) [51], *J. curcas* (59.35%) [52], *M. esculenta* (50.34%) [53], *E. lathyris* (65.87%) [54], and *Triadica sebifera* (64.81%) [55]. Especially, *M. indica* and *M. denticulata* had nearly three times the repetitive sequences of *R. communis*, attributed to long terminal repeat (LTR) retrotransposons in *M. indica* (78.20%) and *M. denticulata* (79.24%) (Fig. 1D). Interestingly, although Gypsy is considered to be the predominant type of LTR in terrestrial plants [56], *M. indica* and *M. denticulata* have conserved large amounts and proportions of the Copia type of LTR since ~0.1–0.3 million years ago (Mya), coinciding with continuous global glaciations (Fig. 1E) [57]. However, their LTR accumulation dynamics differed substantially: *M. denticulata* exhibited moderate, multimodal expansion peaks with Copia curtailment, whereas *M. indica* underwent a recent and precipitous surge of Copia LTR. This suggests that distinct survival pressures drove differential evolutionary trajectories, accelerating and sustaining recent LTRs in *M. indica* versus earlier, phased expansions in *M. denticulata*. Consequently, while LTRs drove genome expansion in both species, *M. denticulata* accumulated and retained to a greater extent various aspects of LTR lineages across multiple evolutionary epochs. This differentiation may be attributed to their divergent phenology and distinct environmental optima, despite their overlapped distribution (same sampling site in this research) (Fig. 1D). Globally, *M. denticulata* exhibited broader latitudinal distribution than *M. indica*, surpassing it in a more southward direction across the equator (Supplementary Fig. S1E and F). This broader latitudinal distribution and diverse LTR conservation in *M. denticulata* reflected stronger adaptations to heterogeneous tropical environments.

Although Euphorbiaceae species are industrially valuable due to their specialized FAs, NA was strikingly rare in this family. Phylogenetic analysis revealed a deep divergence among *Macaranga* species and other NA-producing taxa (*M. oleifera*, *Acer truncatum*, *Xanthoceras sorbifolium*; Fig. 1F), indicating convergent evolution of NA biosynthesis in *M. indica* and *M. denticulata*. From a macro perspective, *M. indica* and *M. denticulata* expanded more than 3000 genes each but *M. indica* contracted more (Fig. 1F; Supplementary Table S10). Both annotated expansion genes and contraction genes in *M. indica* were fewer than in *M. denticulata* (Supplementary Fig. S2A). More specifically, the proportion of lipid metabolism in *M. indica* and *M. denticulata* expanded by 5–25 and 15–40% respectively, while *M. denticulata* contracted by ~5% and *M. indica* scarcely decreased (Supplementary Fig. S2B). These results indicate that *M. denticulata* expanded more genes, especially in lipid metabolism. Notably, *M. denticulata* also evolved unique genes for unsaturated FA biosynthesis, FA degradation, and FA biosynthesis and elongation (Supplementary Table S8), expanding the diversity of lipid compounds and decentralizing metabolic pathways, such as enzyme redundancy, KCS complex modulation, and reciprocal KCS triad regulation. Therefore, the expansion of homologous genes and additional unique genes in *M. denticulata* complicated lipid metabolism, enriching lipid production with the dispersed pathway of certain FAs. The disproportionate expansion and contraction of these gene families in *M. indica* and *M. denticulata* may be linked to divergent LTR retrotransposons, potentially triggered by past climatic changes and shaped species-specific adaptations.

### Active *de novo* fatty acid biosynthesis sustained fatty acid accumulation in both *M. indica* and *M. denticulata* with enhanced efficiency in *M. indica* during seed maturation

**Figure 2:**
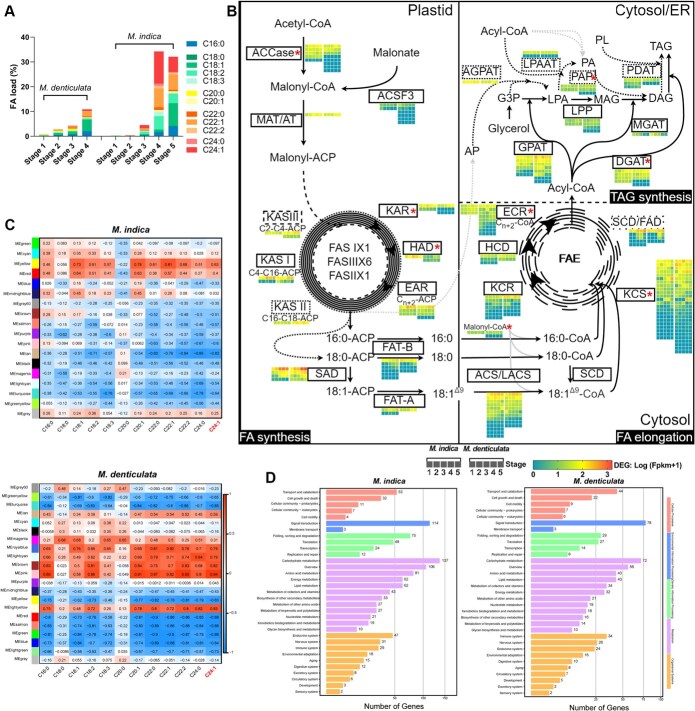
Integrated analysis of FA accumulation and gene expression via WGCNA. (A) Grouped stacked column plots displaying FA content of *M. denticulata* and *M. indica* in four stages (initial fruits were too fragile) and five stages of capsule maturation, respectively; unit = weight ratio (%). (B) Layout heat maps with VLCFA biosynthesis demonstrating three main processes (FA biosynthesis, FA elongation and TAG biosynthesis) with four KEGG pathways: FA biosynthesis (ko00061), FA elongation (ko00062), glycerolipid metabolism (ko00561), and biosynthesis of unsaturated FA (ko01040). Each mosaic presents gene expression by calculating log_10_ fold change of (FPKM + 1). (C) NA–module associations. Heat map depicting correlations between gene modules and NA accumulation levels. (D) KEGG analysis of modules related to NA accumulation. ER, endoplasmic reticulum; ACP, acyl carrier protein; AP, acyl phosphate; CoA, coenzyme A; DAG, diacylglycerol; G3P, glycerol 3-phosphate; LPA, lysophosphatidic acid; MAG, monoacylglycerol; PA, phosphatidic acid; PL, phosphatidylcholine; TAG, triacylglycerol, ACCase, acetyl-CoA carboxylase; ACS/LACS, (long-chain) acyl CoA synthetase; ACSF3, malonyl-ACP synthetase; AGPAT, acyl-glycerol-3-phosphate acyltransferase; DGAT, diacylglycerol:acyl-CoA acyltransferase; EAR, enoyl-acyl reductase; ECR, enoyl-CoA reductase; FAE, fatty acid elongation-related enzymes, including KCS, KCR, HCD, and ECR; FAS, fatty acid synthase; FATA-A/FAT-B, fatty acyl thioesterase A/B; GPAT, glycerol-3-phosphate acyltransferase; HAD, hydroxyacyl-acyl dehydratase; HCD, hydroxyacyl-CoA dehydratase; KAR, ketoacyl-acyl reductase; KAS, ketoacyl-acyl synthase; KCR, ketoacyl-CoA reductase; KCS, ketoacyl-CoA synthase; LPAAT, lysophosphatidic acid acyltransferase; LPP, lysophosphatidate phosphatase; MAT/AT, malonyl-CoA: ACP malonyltransferase; MGAT, monoacylglycerol acyltransferase; PAP, phosphatidate phosphatase; PDAT, phospholipid:diacylglycerol acyltransferase 1; SAD, stearoyl-ACP desaturase; SCD/FAD, stearoyl-CoA desaturase/fatty acid desaturase.

To identify FA biosynthesis genes in *M. indica* and *M. denticulata*, quantitative seed FA analysis revealed that NA accumulated more efficiently in *M. indica* during capsule maturation stages S3 to S5 (Fig. 2A; Supplementary Table S11). Similar to the patterns in other oil-producing plants [58], pseudotime analysis revealed that tetracosenoyl-CoA biosynthesis and NA accumulation in *M. indica* marked an inflection point ending the logarithmic growth phase. This inflection point likely stems from the arrested elongation from C18:1, as indicated by the inverse correlation between elevated C18:1 and reduced C22:1/C24:1 level (Fig. 2A). Through transcriptional analysis, we screened genomic candidates with differential expression: 197 upregulated and 125 downregulated genes in *M. indica*, and 231 upregulated and 146 downregulated genes in *M. denticulata* (Supplementary Table S12), of which 48 and 117 nodes were pathway-specific genes, respectively (Supplementary Table S13). Available RNAs (RNA-seq quality control; Supplementary Table S14) during seed development were quantified at similar levels (Fig. 2B), and significant genes were categorized in four pathways: biosynthesis of unsaturated FA, FA biosynthesis, FA elongation, and glycerolipid metabolism, exhibiting conserved expression patterns and excluding minor duplicated gene subsets. Key nodes of unsaturated VLCFA biosynthesis were constitutively highly expressed in both *M. indica* and *M. denticulata*, such as FAD/SCD or SAD for FA desaturation, ACS/LACS for transport of FA or VLCFA, FAE complex for elongation (Fig. 2B; Supplementary Table S15). Notably, gene expression levels were higher in *M. indica* than in *M. denticulata*, especially with higher expression and fewer orthologous genes, such as *ACCase*s, *KAR*s, *HAD*s, *EAR*s, and *KAR*s in FA synthesis; *ACS*s, *ECR*s, and some *KCS*s in FA elongation; and *GPAT*s, *DGAT*s, *PAP*s, and *LPP*s in TAG synthesis.

We pre-optimized parameters for scale independence and mean connectivity (Supplementary Fig. S3A), and subsequently merged similar modules (Supplementary Fig. S3B). Weighted gene co-expression network analysis (WGCNA) connected each FA and RNA-seq data (Fig. 2C) and iteratively identified 18 and 21 co-expression modules of *M. indica* and *M. denticulata* (Supplementary Fig. S4A). In *M. indica*, one module correlated with NA content (correlation coefficient = 0.63) (Fig. 2C; Supplementary Table S16), while NA accumulation in *M. denticulata* broadly correlated with five proximate modules (correlation coefficient = 0.94, 0.83, 0.82, 0.79, 0.79) (Fig. 2C; Supplementary Table S17), categorizing by KEGG analysis for screening candidate genes and factors (Supplementary Table S18). From one module comparison of *M. indica* and *M. denticulata*, we found that 62 genes and 43 genes were related to lipid metabolism, respectively, indicating that the synthesis and accumulation of specialized FAs correspond to specific individual modules, whereas in *M. denticulata* the multi-module combinations showed mutual interference with limited regulation of individual modules. These multiple modules correlated with NA content in *M. denticulata*, demonstrating that NA biosynthesis and accumulation in *M. denticulata* were regulated by several biological systems, interweaving with other FAs and off-tracking the NA biosynthesis pathway. Interestingly, the NA biosynthesis pathway showed convergent correlation coefficients in *M. indica* (more substrates for NA biosynthesis), indicating tight metabolic channeling toward NA, whereas divergent correlations in five modules of *M. denticulata* led to increased collateral free FAs (Supplementary Fig. S5). From the correlation network, 12 and 17 FAE genes (*KCS*, *KCR*, *ECR*, *HCD*) were concentrated in *M. indica* and *M. denticulata* (Supplementary Table S19), respectively, which constituted a complete biosynthesis pathway in FA elongation. Combined with common regulators, these genes formed a more complex network in *M. denticulata*, which contained more nodes representing FAEs and 1.5–15 times more edges (representing correlated genes) connected to them compared with the network in *M. indica* (Supplementary Table S19, Supplementary Fig. S4B). Furthermore, this network in *M. denticulata* was significantly enriched in lipid metabolic processes, such as lipid transformation, degradation, and TAG synthesis, whereas the network in *M. indica* showed stronger associations with FA biosynthesis, plant hormone pathways, and Ca^2+^-dependent stomatal closure. Furthermore, in *M. denticulata*, FAEs correlated more with downstream metabolic process such as lipid transformation, degradation, and TAG synthesis, while in *M. indica* they were associated with upstream biosynthesis and environmental responses including FA biosynthesis, plant hormone pathways, and Ca^2+^-dependent stomatal closure (Supplementary Table S20, Supplementary Fig. S4B). These patterns reflected divergent metabolic strategies for VLCFAs: they were continuously synthesized from long-chain FAs in *M. indica* but were rapidly utilized in *M. denticulata*.

Integrated evidence from gene expression, expansion, co-expression networks, and module analysis indicated that *M. indica* simplified and fortified the VLCFA biosynthesis pathway. Conversely, *M. denticulata* employed a more complex regulatory strategy, involving multiple modules and more regulatory elements to engage in diverse lipid turnover processes, likely occurring at the expense of high output in any one particular FA and redirecting metabolic flux toward a wider variety of lipids.

### Fatty acid elongation synergy blueprinted subtle differences in the VLCFA biosynthesis pathway

FAE and FAD independently regulated VLCFA biosynthesis, with FAD determining desaturation and FAE controlling chain length. In *M. denticulata*, the accumulation of octadecanoic acid suggested an interruption at the initial elongation step, although no distinguishable differential expression was detected in the core biosynthetic enzymes. Genomic analysis revealed that *KCR*s and *ECR*s formed clusters on chromosomes 1 and 2, respectively. Despite a minority (2/6 *KCR*s, 3/7 *ECR*s) in *M. denticulata* remaining unassembled, *ECR* paralogs constituted a second cluster with equivalent expression levels during capsule maturation. In contrast, *M. indica* employed a more direct and efficient strategy, featuring only one gene cluster whose expression dynamics closely paralleled the NA accumulation curve (Supplementary Fig. S6, Supplementary Table S15).

Generally, KCSs served as the rate-limiting enzyme for FA elongation in specialized tissues and functioned to diversify products by substrate selectivity. In *M. indica* and *M. denticulata*, *KCS* genes were dispersed across all chromosomes, with the exception of a subset of *KCS11* paralogs that formed a partial gene cluster on chromosome 10. However, phylogenetic analysis revealed that the KCS11 cluster was rearranged from KCS11 and KCS11/20. Interestingly, the additional cluster in *M. denticulata* appeared to have arisen through an inverse duplication event of a genomic segment that co-integrated upstream and downstream paralogous genes, with some structural discrepancies (Supplementary Fig. S7). Since the vast majority of KCSs in *M. indica* and *M. denticulata* clustered within the Euphorbiaceae clade, the duplication events and insertions were inferred to have occurred more recently. Notably, genes falling outside of the Euphorbiaceae clade were closer to Convolvulaceae, Fagaceae, Cruciferae, etc., including one shared KCS4 ortholog, one KCS12 ortholog, one MdKCS21, and one MiKCS7 (Supplementary Fig. S7).

Through MEME Suite analysis combined with phylogenetic examination of gene completeness (Supplementary Fig. S8A), a clear dichotomy among KCSs was revealed based on the presence or absence of the MEME9 motif (the most conserved sequence with LKYVKLG) in the N-terminal region. Those containing MEME9 were associated with the major function of FA elongation [59, 60], exhibiting distinctly higher gene expression during capsule maturation. Pairwise alignment between *M. indica* and *M. denticulata* further showed that KCS7s and KCS11s retained more ancestral sequence fragments while MEME5/6/7 displayed higher frequencies of mutation. Conversely to gene expression in capsule maturation, certain *KCS*s, including *KCS15*, *KCS6*, *KCS10*, and *KCS19*, displayed stronger expression in male tissues and leaves of *M. denticulata*, underscoring the importance of VLCFAs in the vegetative growth of this species.

Domain prediction revealed that most MiKCSs and MdKCSs contained two characteristic domains (Supplementary Table S21): FAE1/Type III polyketide synthase-like (IPR016039), which catalyses substrate condensation during FA elongation, and *β*-ketoacyl-[ACP] synthase III, C-terminal (IPR013747), involved in mediating heterodimer formation [61]. Comparative analysis of residues located at four functional sites – the dimer interface (DI), the product-binding site (PBS), the malonyl-CoA binding site (MCBS), and the active site (AS) – showed that amino acid substitutions in KCS proteins induced conformational shifts or altered solvent accessibility in their tertiary structures. Structural analysis revealed that even orthologs located in similar chromosomal regions employed distinct structural templates, as observed in KCS5, KCS7, KCS15, etc. (Supplementary Table S22), despite their close phylogenetic relationship. Each KCS protein contains a conserved catalytic AS triad (N-H/Y/P-C), accompanied by 9–11 PBS, 7–8 MCBS, and 30–31 DI residues, exceeding previous estimates in both number and complexity. Owing to more extensive *KCS* gene duplications in *M. denticulata*, further analysis suggests its orthologs may exhibit shared, unique, or more diverse structural templates, ultimately resulting in greater structural divergence among KCS proteins compared with those in *M. indica* (Supplementary Table S22).

In summary, compared with *M. indica*, *M. denticulata* exhibited a more complex FAE system characterized by an increased number of *ECR* gene clusters, additional *KCS* duplications, multiple copies of key and redundant genes, and greater structural diversity among KCS enzymes. These features collectively enhance the regulatory capacity and catalytic product diversity of the FAE pathway.

### Stable environmental conditions and specific biosynthesis pathway enhanced VLCFA biosynthesis in *M. indica*

To enhance FA elongation efficiency, coordinated expression of the FAE enzyme complex is essential with synchronized transcriptional activation of its individual components maximizing FAE activity. Analysis of FAE promoter architectures revealed that *M. indica* and *M. denticulata* have evolved distinct environmental adaptation strategies. Paralogous *KCS* genes often exhibited varied combinations of interlaced *cis*-regulatory elements (CREs), contributing to broader environmental responsiveness (Supplementary Table S23). For example, *MiKCS4* and *MiKCS4 like* (0/1 cold-related CREs) (5/0 box-4) versus *MdKCS4* and *MdKCS4 like* (0/1 cold-related CREs) (5/2 box-4), *MiKCS12* and *MiKCS12 like* (1/0 wound response) versus *MdKCS12* and *MdKCS12 like* (0/1 response). Paralogous *KCR*, *HCD*, and *ECR* genes showed low CRE overlap between *M. indica* and *M. denticulata*, while differences in KCS were mainly quantitative (Supplementary Fig. S8B). Overall, *M. indica* exhibited a reduction in CRE number, density, and promoter proximity (Supplementary Fig. S9A). Notably, *MiECR* promoters lacked CREs for cold, differentiation, secondary metabolism, and drought, and had fewer distal light and TF motifs, yet all *MiECR* genes were highly expressed and correlated with NA content.

Furthermore, based on connectivity to NA content, 12 KCS sequences, 1 KCR sequence, 2 HCD sequences, and 4 ECR sequences were detected in *M. denticulata*, and 8 KCS sequences, 1 KCR sequence, 1 HCD sequence, and 2 ECRs sequences were detected in *M. indica*. Consistent with previous analyses, of the highly correlated proteins, only MiKCS12 and MdKCS19d2 lacked the MEME9 domain. WGCNA revealed that only *M. denticulata* contained two multigene nodes associated with reproductive growth, which were correlated with two more *KCS* gene nodes (Supplementary Fig. S9B). Functional annotation indicated that *M. indica* is strongly linked to cold stress and phosphorus starvation (Supplementary Table S24), matching its maturation period from November to February and the terrestrial phosphorus limit environment. This association was particularly supported by gene pairs with cold stress – *DREB*–*MiKCS11d*, *DREB1A*–*MiKCS11b*, *DREB1F*–*MiKCS11b* [62] – and phosphorus starvation – *MiKCS11c*–*PHR1*–*HCD*_p2 [63], *PSR1*–*MiKCS11c* [64]. In contrast, *M. denticulata* exhibited more abiotic stress-related genes scattered across environmental factors, suggesting VLCFA biosynthesis contributed broadly to environmental adaptation.

### Fatty acid metabolic fate underlaid VLCFA divergence in *M. indica* and *M. denticulata*

Given the differential metabolic flux of FAs across developmental stages [37], we quantified free FA levels during capsule maturation (Supplementary Fig. S5) through integrated comparison with total FA profiles (Fig. 2A), mirroring total FAs and revealing that free VLCFAs were predominantly synthesized and rapidly converted during late maturation stages. In contrast to the analogous accumulation patterns of NA precursor (C16:0, C18:0, C18:1, C20:1) in both *M. indica* and *M. denticulata* (Fig. 2D), erucic acid and NA biosynthesis in *M. denticulata* stagnated during the rapid NA biosynthesis phase. Furthermore, branch-pathway products including C16:1, C18:3, and C20:2 accumulated to significantly higher levels in *M. denticulata* relative to *M. indica*. This shift provided abundant substrates of free VLCFAs, attributable to coordinated actions of FAE biosynthesis, degradation, or transformation.

**Figure 3:**
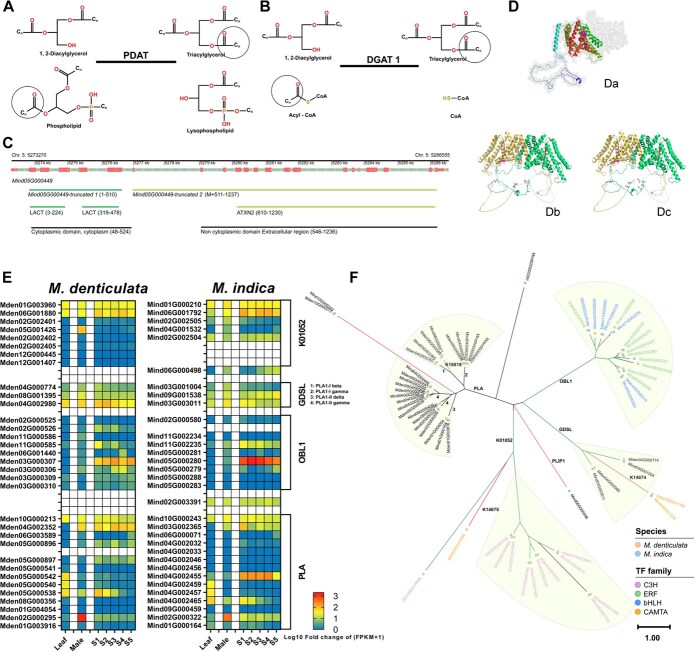
Structure analysis of DGAT and PDAG and expression and phylogeny of TAG lipases in *M. indica* and *M. denticulata*. (A) PDAT mechanism: catalytic transfer of acetyl group to TAG sn-3 position from phospholipid (black circle). (B) DGAT1 mechanism: acetyl transfer to TAG sn-3 from acyl-CoA (black circle). (C) Mind05G000449 (PDAT) architecture: exons (red bars); translation direction (white arrows). Underlying lines denoted the truncations, motifs, and subcellular localization. (D) DGAT dimer configurations: heterodimer (Mden07G000505-Mden12G000061; cartoon/dot surface) and homodimers (Mden07G000505-Mden07G000505, Mind07G000461-Mind07G000461; cartoon, lateral view). (E) Expression clustering: heat map showing mean expression of TAG lipases/isozymes. (F) Phylogenetic analysis: radial unrooted tree with terminal branches color-coded by species (*M. denticulate*, orange; *M. indica*, blue). Genes were annotated by TF family (colored text). Divergent branches (red) indicate evolutionary distance from core lineages (green). Clades with shared functional annotation are highlighted with light yellow leaf-shaped backgrounds.

To boost NA content in *Macaranga*, enhancing TAG biosynthesis was crucial for NA accumulation. Plants preferentially incorporated VLCFAs at the sn-1/3 positions of the glycerol backbone, while reserving the sn-2 position for shorter-chain FAs (C16–C18) through two conserved pathways: the DGAT-mediated Kennedy pathway and the PDAT-driven phospholipid remodeling pathway (Fig. 3A and B).

*Macaranga indica* and *M. denticulata* possessed three and two PDATs, respectively (Supplementary Fig. S10A). Lecithin:cholesterol/phospholipid:diacylglycerol acyltransferase (LACT, IPR003386) represented the core catalytic domain of PDAT enzymes. All MiPDATs and MdPDATs contained one or two LACT domains (Supplementary Table S25). However, one MiPDAT (Mind05G000449) exhibited persistently high expression and contained a structural fusion with a perianth-like domain harboring an ataxin-2 (ATXN2, IPR045117) motif (Fig. 3C, Supplementary Fig. S10B). This fusion naturally formed a mosaic architecture, in which two RNA-binding Sm-like domains (LsmAD, IPR009604) and an AD domain terminus clustered adjacent to this unique structure, but remained non-contacting with it. This spatial arrangement may sterically hinder target proteins, and localize to divergent organelles (Fig. 3C), potentially impairing PDAT enzymatic activity in *M. indica* and decreasing the PDAT-driven pathway.

Recent studies indicated that atypical FA accumulation as TAG is primarily catalysed by DGATs, with DGAT2 exhibiting specific substrate preference for NA [65, 66]. Phylogenetic analysis of acyltransferases, including DGAT, PDAT, wax ester synthase/diacylglycerol acyltransferase (WSD) and DGAT1/2-independent storage lipid synthase (DIESL), showed that DGATs diverged into three primary clades (DGAT1, DGAT2, DGAT3), while WSD, PDAT, and DIESL clustered within a transitional zone divided by non-vascular plant homologs (Supplementary Fig. S11). In contrast to DGATs from *M. oleifera* (high NA) and *Camelina sativa*, *Macaranga* DGATs phylogenetically clustered in the clade of Euphorbiaceae. However, MiDGAT3 and MdDGAT3 occupied positions marginal to MoDGAT3, suggesting distinct selectivity in FA incorporation in TAG. Intriguingly, only some acyltransferases of *M. denticulata* clustered phylogenetically within the non-vascular plant clade, suggesting either retention of ancestral gene duplications or horizontal gene transfer from algal symbionts in *M. denticulata*. Furthermore, all identified exogenous *M. denticulata DGAT*s/*PDAT*s (*MdPDAT*, *MdDGAT1E*, and *MdDGAT2E*) lacked TATA-box promoters (Supplementary Table S26) and showed no detectable expression in leaves or in fruits during capsule maturation, strongly supporting their origin via horizontal gene transfer. Besides, these three genes harbored unique CREs with partially interconnected intergenic regions; for example, *MdDGAT1E* and *MdDGAT2E* shared an RY element [67] mediating during late seed maturation and early germination (Supplementary Table S27), impeding the function of core DGAT and TAG accumulation in a critical period. Furthermore, drawing on DGAT1 methodologies [68, 69], DGAT models of *M. indica* and *M. denticulata* were constructed and adopted a funnel-shaped structure formed by at least seven transmembrane helices (TMs) (Supplementary Figs S10C and S12, Supplementary Table S27). The absence of TM1 [70] abolished the enzymatic activity of MdDGAT1E (Fig. 3D). Given that DGAT1 typically functions as a dimer, the capacity of MdDGAT1 and MdDGAT1E to form both homodimers and heterodimers likely resulted in competitive inhibition of MdDGAT1 catalysis (Fig. 3E). Functional structure check verified the completeness of TMs, including the following: (i) the important catalytic core, histidine–glutamic acid (H449–E450) in TM7 for facilitating its nucleophilic attack on the thioester bond of acyl-CoA; (ii) concatenated structure of dimer by TM5, TM6, and TM9 (Fig. S10C); and (iii) a substrate-binding pocket interacted with the CoA moiety via L508, Q504, W368, F375, and S445 of TM6–9 (Fig. S10D). Although DGAT1 was rather conserved in *M. indica* and *M. denticulata*, mutation sites occurred at structural pivot points (MiDGAT1: S311–R388 versus MdDGAT1: G311–C388) within TM5 and TM6, likely expanding the spatial dimensions of MiDGAT1 and favoring longer-chain FAs (Supplementary Fig. S10E, Supplementary Table S28).

Dynamic combinatorial RNA-seq and KEGG analysis revealed that TAG lipases and isozymes negatively regulated TAG accumulation (Fig. 3E; Supplementary Table S29). These enzymes were classified into four functional clusters in *M. indica* and *M. denticulata*: oil body lipase 1 (OBL1), GDSL-type esterase/lipase, lysosomal acid lipase/cholesteryl ester hydrolase (K01052 type, LIP1/2), and phospholipase A1 (PLA1) (Fig. 3F). Most TAG lipases exhibited low or declining expression during capsule maturation, especially at stage 3, coinciding with the onset of NA accumulation such as PLA1-II γ. In contrast to TAG biosynthesis, TAG lipase (outside K01052) expanded slightly more in *M. indica* than *M. denticulata*, resulting in a reduction in multiple lipids and promoting the accumulation of major lipids. Conversely, regulator genes were more numerous in *M. denticulata* than *M. indica*, suggesting a more complex regulatory mechanism (Supplementary Table S30).

Collectively, using analysis of free and storage FAs, *M. indica* strengthened VLCFA storage in FA metabolic processes. In contrast to *M. denticulata*, *M. indica* upregulated TAG biosynthesis via the DGAT pathway, while concurrently promoting the degradation of other transient lipid storage, shifting results toward a composition in which specific FAs were more prominent.

### Differential evolutionary trajectories of *M. indica* and *M. denticulata* via chromosomal rearrangements and gene duplications

**Figure 4:**
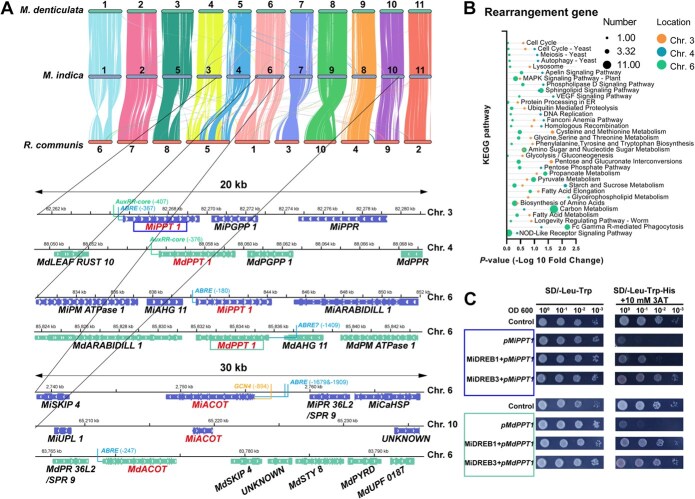
Gene rearrangement of chromosome 3, 4, and 6 in *M. indica* and *M. denticulata*. (A) Synteny analysis among *M. indica*, *M. denticulata*, and *R. communis*. Chromosomes/scaffolds (bars); syntenic blocks (lines). Genomic rearrangement schematic: FA biosynthesis genes showing mRNA structure (blue rectangles: exons [wide]/introns [narrow]; translation direction [white arrow]). Candidate genes (<15 kb; black text) and target genes (red text) are annotated. CREs scaled proportionally: auxin-related (green), gibberellin-associated (blue), endosperm-specific (orange). (B) Rearrangement hotspot mapping: triaxial bubble plot of KEGG-enriched genes on chromosome 3 (red), chromosome 4 (blue), and chromosome 6 (green); bubble size = gene dosage. (C) MiDREB1 and MiDREB3 bind to promoter DNA sequence of MiPPTChr.3 and MdPPTChr.6. Control = pGBKT7-p53 + pGATD7-T.

Interspecies collinearity analysis revealed that 28.30 and 30.83% of genomic regions were linked within collinear blocks in *M. indica* and *M. denticulata*, respectively (Fig. 1B). Within these blocks, 1881 pairs of collinearity blocks (Supplementary Table S31) underpinned the high-identity genomic configuration (Fig. 4A; Supplementary Fig. S13A). On the whole, homologous genes in two chromosomes (chromosomes 4 and 5 in *M. denticulata* and chromosomes 3 and 4 in *M. indica*) seem spliced and conjugated alternately into one chromosome with correspondence to *R. communis*. Fourteen, 8, and 119 genes were translocated to fragments of chromosomes 3, 4, and 6, respectively (Fig. 4B). Also, four recombinant gene permutations functioned in the FA biosynthesis pathway (Supplementary Table S32): MiACCase catalysed the initial conversion of acetyl-CoA to malonyl-CoA, while palmitoyl-protein thioesterases (MiPPT1^Chr.3^ and MiPPT1^Chr.6^) and an acyl-CoA thioesterase (MiACOT) hydrolyse FA-CoA to release free FA. Notably, MiPPT1 acted specifically on short-chain FAs [71], while MiACOT processed VLCFAs [72]. Furthermore, recombination blocks generated tandem gene arrays within a 10-kb region in *M. indica* and *M. denticulata* (Fig. 4A), forming ABA-sensitive clusters especially in *M. indica*, including *MiPPT1*^Chr.3^–*MiPGPP1*–*MiPPR*, and *MiPMATPase1*–*MiAHG11*–*MiPPT1*^Chr.6^–*MiARABIDILLO1*, and *MiSKIP4*–*MiACOT*^Chr.6^–*MiPR36L2/SPR9*–*MiCaHSP*, which assimilated into gene clusters sensitive to abscisic acid (ABA) or related abiotic stress via coessential CREs; for example, *AHG11*, considered a cytoplasmic ABA receptor [73]; and *ARABIDILLO1* regulated by ABA [74] and ABA related CREs (ABREs) [75]. Unexpectedly, three linkages resulted in optimizations or divisions in FA biosynthesis in *M. indica* compared with *M. denticulata*: an endosperm-localizing GCN4 motif [76] upstream of *MiACOT*, and an additional ABRE at the 5′ end of *MiPPT1* on chromosome 3 and *MiACOT* on chromosome 6. However, homologous genes of *M. denticulata* weaken the ABA regulation, e.g. absence of (*MdPPT1*^Chr.4^) or the elongation distance of ABRE (*MdPPT1*^Chr.6^) and GCN4 (*MdACOT*^Chr.6^). Through yeast one-hybrid assays (Fig. 4C), we found that MiDREB1 and MiDREB3 of DREB family were highly correlated with NA accumulation, showing a certain degree of selective interaction with the *MiPPT1* promoter. Interestingly, while no corresponding *DREB* genes were associated in *M. denticulata*, MiDREB1 and MiDREB3 both demonstrate strong interactions with the *MdPPT1* promoter. The above results indicate that cold-associated DREB transcription factors play a definitive role in FA biosynthesis in *M. indica*, whereas their function in *M. denticulata* remains less certain. Nevertheless, we cannot exclude the possibility that transgenic expression of *MiDREB1* or *MiDREB3* in *M. denticulata* could enhance FA biosynthesis in its seeds.

In both species, dispersed duplications and WGD/segmental duplications accounted for the majority of duplicated genes (Supplementary Table S33), analogous to some abiotic stress-tolerant plants and oil crops [77]. Duplication retention, biased toward ancient dispersed duplications [78, 79], was filtered by mechanisms including the acquisition of new functions, partial redundancy, or CREs. Therefore, tandem and proximal duplications facilitated environmental adaptation through natural selection and contributed to the production of special metabolites. Contrary to previous understanding, *M. indica* harbored ~3-fold more proximal duplications in lipid metabolism compared with *M. denticulata* (Supplementary Table S34), likely underlying its rapid functional divergence in tissue specificity, via neofunctionalization or subfunctionalization of ancestral genes. Overall, *M. denticulata* exhibited more extensive gene duplications, leading to a larger genome size and enhanced adaptation to austere environments, whereas *M. indica* maintained a simpler metabolic profile while expanding its regulatory system. To unveil key environmental factors polarizing evolutionary trajectories, *K*_a_/*K*_s_ analysis (ratio of non-synonymous to synonymous mutations) highlighted seven *M. indica* genes and nine *M. denticulata* genes under positive selection, respectively (*K*_a_/*K*_s_ > 1.0), indicating that these idiosyncrasies (adaptability to stress and plant phenology) were caused by natural or artificial selection (Supplementary Table S35). Among the selected genes, two transcription factors, *MiVRN1* (vernalization or flowering pathways [80]) and *MdbHLH84* (drought resistance or autoimmunity [81, 82]) were highlighted in this selection (Supplementary Fig. S14). These functional divergences drove distinct phenological adaptations: generative propagation in *M. indica* and vegetative growth in *M. denticulata*.

### Structural variants (SNPs/indels/presence–absence variations) enrich the gene pool of *M. indica* and mitigate inbreeding depression

**Figure 5:**
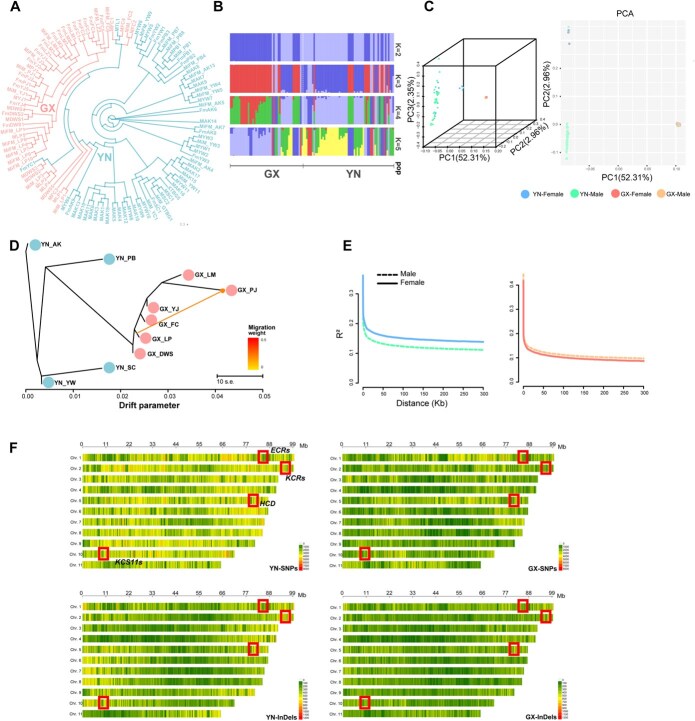
Genome level analysis of *M. indica* from different populations. (A) Circular dendrogram of *M. indica* by resequencing demonstrated two distinct clusters: one from Yunnan Province (YN), and another combining Guangxi Autonomous Region and Guizhou Province (GX). Samples from YN=blue, GX=red. (B) Model-based clustering of *M. indica* with *K* = 2–5. (C) 3D and ichnographic scatter plots showed genotype clustering of *M. indica* from YN and GX, highlighting sequence variation independent of sex. (D) Maximum likelihood phylogeny showing a migration event (orange arrow) within GX populations. All GX samples were clustered at the branch of YN_PB. Scale bar shows the average standard error (s.e.) of the entries in the sample covariance matrix. Red dots and blue dots trace the sample from GX district and YN district, respectively. (E) Curves show LD decay of *M. indica* for male and female from YN and GX; pairwise *r*^2^ values indicate linkage equilibrium of two genes in *X*-axis distance. Solid line curve=Female, dotted curve=Male, blue and green curve=YN, red and yellow curve=GX. (F) Genetic diversity of *M. indica* populations in YN and GX populations. Heat map-style bar plots comparing SNP or indel densities across the genome, with minimum bin size of 0.1 Mb.

Despite being isolated in distinct habitats but exhibiting asynchronous flowering among sympatric *Macaranga* species, the undomesticated dioecious species *M. indica* and *M. denticulata* maintained considerable heterozygosity. The genomic heterozygosity rates for *M. indica* and *M. denticulata* were estimated at 0.75 and 0.65%, respectively (Supplementary Table S1), with two obvious peaks in the distribution. Resequencing 110 individuals of *M. indica* from approximately the same latitude (Supplementary Tables S36 and S37) with an average depth of 19.61 (Supplementary Table S38), revealed a mean 13.05 M SNPs and 1.29 M indels (Supplementary Table S39). Both SNP and indel densities in *M. indica* exhibited significant geographic variation, yet showed minimal divergence among individuals from the same populations (Supplementary Table S39). Phylogenetic analysis (Fig. 5A), ADMIXTURE plots (*K* = 2–5; Fig. 5B), and PCA (Fig. 5C) collectively demonstrated that *M. indica* comprised two evolutionarily distinct groups with significant SNP/indel differentiation (Supplementary Table S40): a Yunnan (YN) clade and a Guangxi–Guizhou (GX) cluster (Supplementary Table S41). Treemix analysis positioned the sample YN_PB as an intermediate node between the YN and GX populations (Fig. 5D). Additionally, a single short-distance migration event was detected from GX_LP to GX_PJ. Geographically, YN_PB bridged the YN and GX populations (Supplementary Fig. S15A), with its nucleotide diversity intermediate between the two groups. This pattern indicates that gene flowed from YN to GX populations, where YN_PB functioned as a genetic stepping-stone. SNP distribution visualized at 0.1 Mb resolution (Supplementary Fig. S9F) revealed higher SNP density in YN populations of *M. indica*. Concurrently, concentrated clustering along PC2 in GX populations (Fig. 5E) indicated the enhanced local adaptation traits. Comparative analysis of nucleotide diversity between the two *M. indica* clusters further corroborated higher genetic diversity in YN populations. Additionally, population divergence (*F*_ST_; Supplementary Fig. S15B) and Tajima’s *D* values (Supplementary Fig. S15B) revealed distinct evolutionary trajectories: GX populations showed signatures of recent population contraction (*D* > 0), while YN populations maintain mutation–drift equilibrium (*D* ≈ 0). In our common garden cultivation for *M. indica*, accessions from GX exhibited a 2-year shortening of the juvenile phase, accelerating reproductive cycles and enhancing adaptive evolution. The pioneer species traits further amplified genetic differentiation through natural selection, leading to elevated genetic drift within GX populations. Additionally, at comparable altitudes, GX populations were distributed further north than YN populations. This latitudinal divergence exposed them to distinct environmental factors (e.g. cooler thermal conditions) that directly influenced *Macaranga* population dynamics.

Moreover, linkage disequilibrium (LD) decay in *M. indica* occurred more rapidly with less physical distance than some plants, e.g. soybean [83], indicating limited artificial domestication (Fig. 5E). Notably, higher *r*^2^ values in female vs male *M. indica* at equivalent paired distances reflected sexual dimorphism. Coupled with prolonged breeding cycles, this pattern precluded intense artificial selection or rapid domestication, suggesting reduced future risk of inbreeding depression [83]. Consequently, hybridization between YN and GX populations held significant promise for *M. indica* breeding. Furthermore, sexually dimorphic LD decay patterns (Fig. 5E) indicated that YN accessions demonstrated superior paternal fitness in inter-population crosses.

To evaluate genomic distinctiveness, we compared *M. indica*, *M. denticulata*, and *R. communis*, annotating 890, 1446, and 478 unique genes, respectively (Fig. 4B). Using pan-genome comparative analysis, we identified 9076 and 3315 presence–absence variations (PAVs) from *M. indica* vs *M. denticulata* and *M. indica* vs *R. communis*, respectively (Supplementary Fig. S13C, Supplementary Table S42). This substantial PAV divergence primarily stemmed from differential retention of homologous genes among congeneric species. According to KEGG analysis, *M. denticulata* exhibited more distinct genes (Supplementary Table S43) and unique genes (Supplementary Table S44) in lipid metabolism, likely decentralizing synthetic pathways or elevating flux thresholds in specific FA biosynthesis routes. In contrast, *M. indica* enriched more genes related to unique environmental adaptation, including circadian rhythm, plant–pathogen interaction, and major environmental information processing pathways.

The results showed that the risk of inbreeding depression or genetic bottlenecks in *M. indica* was manageable or negligible, supported by luxuriant wild germplasm resources with adequate diversity in SNPs, PAVs, and inversions (Supplementary Table S45). Thus, subsequent domestication efforts can strategically focus on enhancing FA biosynthesis and accumulation.

## Discussion

### Fatty acid synthesis and storage related to environment adaption

This study presents the first high-quality *de novo* genome assemblies of *M. indica* and *M. denticulata* (Euphorbiaceae). Like other woody Euphorbiaceae species, *M. indica* and *M. denticulata* harbored abundant repetitive sequences, enhancing genomic and phenotypic diversity. Integrative analyses revealed distinct evolutionary trajectories: *M. indica* retained enhanced genetic capacity for physiological processes (notably VLCFA biosynthesis), while *M. denticulata* had accumulated adaptive genes supporting broader geographic distribution via more ancient gene expansions. This adaptability correlated with increased FA metabolic flux rather than TAG storage in *M. denticulata*, and correlated with the more complex pathway and homologous genes but lower expression levels. Based on comparison of phenological traits with *M. denticulata*, *M. indica* bred in cooler conditions and inhabited higher elevations, requiring abundant and specific TAG storage for germination. We suggest that *M. indica* seeds likely possessed a chilling requirement correlated to ABA [84], supported by the presence of more CREs and their closer association with ABA-related rearrangements in this species. These findings address a gap in seed research of tropical plants at high altitudes. However, the limited sampling in this study constrained the assessment of *M. indica* genetic diversity and domestication potential, necessitating future validation with broad sampling.

### Key transcription factors and critical genes await further exploration

In this study, we also identified transcription factors and stress-related genes associated with FA biosynthesis and environment response. Although most genes exhibited low or undetectable expression, several predicted upstream regulators showed traceable activity. Notably, *ASIL1* (arabidopsis 6b-interacting protein 1-like 1, a trihelix transcription factor) was highlighted. *ASIL1* represses LEC1/LEC2 by binding GT elements and overlapping G-boxes. Crucially, suppression of *ASIL1* reduced TAG accumulation and compromised tolerance of salt, drought, and cold stress. Here, *ASIL1* exhibited consistently high expression during capsule maturation in both *M. denticulata* and *M. indica*, suggesting a metabolic engineering strategy to enhance FA biosynthesis, since silencing *ASIL1* in mutant lines would derepress downstream targets (LEC1, LEC2, OLEOSIN) and amplify lipid accumulation [85]. Regarding phosphate starvation, we identified two genes strongly correlated with FA elongation in *M. indica* (Supplementary Fig. S9B): *MiPHR1* (linked to *MiKCS11c* and *MiHCD_p2*) regulated lipid remodeling and TAG accumulation, and *MiPSR1* (associated with *MiKCS11c*) may modulate similar pathways. Concurrently, elevated *HDA19* expression, a key regulator of phosphate stress response and lipid restructuring in *M. indica* [86], underscored the critical role of phosphate limitation in TAG biosynthesis across *Macaranga* species (Supplementary Table S30). Under cold stress, transparent testa (TT) transcription factors TT2 and TT8 typically suppressed FUS3 (TAG biosynthesis) and FAE1 (FA elongation). However, neither TT2 nor TT8 was identified in this study. Instead, most TT12 homologs regulated pigment/color compounds [87], a pattern consistent with the shift in *M. indica* episperm color during capsule maturation. These findings suggest that neither species modulates FA biosynthesis via TT-mediated chilling responses. For heat shock (32–50°C, optimum 42°C) [88], TAG accumulation occurred independently to TT related pathway. Despite *M. denticulata* experiencing higher capsule maturation temperatures (Supplementary Fig. S1D), insufficient *MdPDAT* expression/copy number resulted in TAG deficiency.

We also examined the regional genotypic disparities of *M. indica* in China. It is postulated that the Guangxi populations may have diverged from some populations of Yunnan, through environmental adaptation to their new habitat, suffering a loss of diversity. Remarkably, under the controlled conditions of a common garden, certain Guangxi individuals manifested traits indicative of adaptation to northern migration (data not shown), such as a markedly abbreviated juvenile phase, dwarfed stature, and condensed inflorescences, a syndrome analogous to that of *R. communis* [89, 90]. The central uncertainty lies in whether these phenotypic changes represent a direct adaptive response. Here we put forward a bolder hypothesis: *M. indica* could be domesticated from a perennial woody plant to become an annual herbaceous form similar to *R. communis*.

NA biosynthesis specifically depended on FAE activity, with screening parameters encompassing (i) NA production efficiency, (ii) TAG assembly capacity, (iii) environmental responsiveness, and (iv) transcriptional regulation. As NA biosynthesis required erucic acid as substrate, optimizing FAE activity, substrate specificity, and enhancing TAG biosynthesis represented the critical engineering targets for NA biosynthesis. Notably, wounding, low temperature, and P starvation are strongly correlated with NA production in *M. indica*, concurrently activating ancillary pathways including lipid transport, FA desaturation, and hormone-mediated regulation. However, *M. indica* (predominantly tropical) exhibited NA biosynthesis stimulation under mild temperature drops rather than extreme cold stress, and the response aligned with Brassinolide (BR)-dependent FA elongation and associated stomatal regulation. We therefore propose targeting moderate temperature shifts as optimal elicitors and other candidate known elicitors (drought, phosphate limitation, and wounding) for NA biosynthesis in this research, but we highlighted the habitat adaptation specificity of *M. indica*: sensitivity to cooler thermal conditions, seasonal drought, and submergence stress. As crop yields alter under global climate change, adaptation mechanisms to the tropical environment of *Macaranga* provide potential breeding directions for cultivation, especially in dicotyledonous woody plants. Intriguingly, despite some elevated expression and gene copy numbers of FA biosynthetic genes in *M. denticulata*, NA accumulation remained constrained, suggesting post-transcriptional regulation (e.g. translational efficiency or enzyme kinetics). While searching for and validating transcription factors that regulate *MiKCS11c* and *MiKCS11d*, we discovered that WRINKLED1 (WRI1) bound effectively to their promoter regions, although the specific interaction mechanisms require further investigation (Supplementary Fig. S16). Therefore, future work would be integrated in proteomics and epigenetics via ATAC-seq, CUT and Tag, iTRAQ, and 4D Label-free to resolve these mechanisms.

### Domestication potential and application value of *M. indica*

With climate warming, we have also observed that the geographical distributions of *M. indica* can shift further north. In sympatric distributions, *M. indica* tends to inhabit higher elevations, and shows a tendency to migrate northward in China. This is similar to other Euphorbiaceae plants such as tung oil tree, castor bean, and Chinese tallow tree. To adapt to colder regions, these plants have developed traits such as high oil content, enabling their seeds to survive cold winters. Based on our lipid analysis and migration patterns, it is possible for *M. indica* to be distributed or introduced further north. More notably, seeds from Guangxi Autonomous Region in the common garden exhibited a shortened juvenile phase. This resembles the domestication process of castor bean over thousands of years; it evolved from a woody plant in Africa to a herbaceous form in Asia, Europe, and the Americas [89]. The *M. indica* populations in China appear to be undergoing a similar process. Given the close genetic relationship between castor bean and *Macaranga*, this observation may reveal mechanisms underlying the evolution of woody plants toward herbaceous forms or providing potential for shortening of breeding cycles in woody plants. As for changes in seed oil content, more data from *Macaranga* populations in higher latitudes and long-term stable transplantations in homogeneous gardens are needed.

Beyond evolutionary significance, *Macaranga* conservation demands urgent attention. Currently, only 18.23% of described species (181 taxa as of May 2023) are documented in human use or trade (Supplementary Fig. S1E, Supplementary Table S46), with 25% facing high extinction risk amid accelerating population declines (IUCN 2023). Most species served only as firewood/timber, despite documented non-traditional applications including pharmaceutical compounds, the *Macaranga*-type Pacific propolis [91], and flavored beverages [92]. Rational utilization remains critically limited. This study advocates *Macaranga* valorization as a sustainable source of NA, proposing bioprospecting as a strategy to incentivize species conservation.

### Conclusions

**Figure 6:**
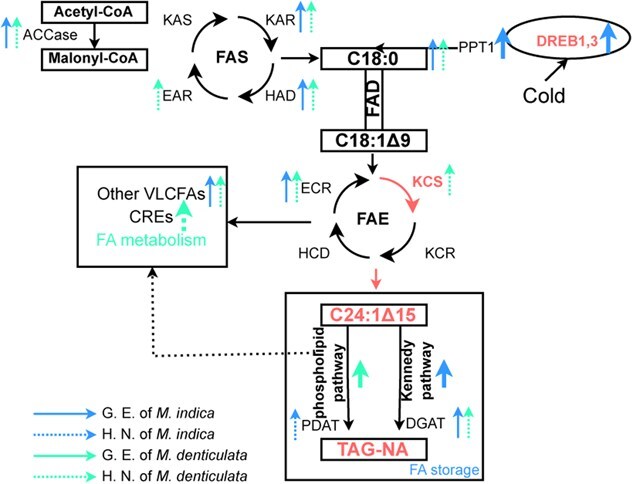
Schematic comparison of *M. indica* and *M. denticulate*. A simplified flowchart illustrating differential gene expression or compound contents, and gene diversity or compound percentage, and regulatory mechanisms between *M. indica* and *M. denticulata*. Red and green colors denote respective increases and decreases in diversity and relative levels. G.E., gene expression or compound content; H.N., gene diversity or compound percentage.

In this study we assembled the genomes of *M. indica* (912.80 Mb) and *M. denticulata* (955.90 Mb), annotating 33 246 (87.56%) and 35 836 (88.51%) protein-coding genes, with repetitive sequences comprising 81.82 and 79.31%, respectively. LTRs dominated these repeats, exhibiting an unusually high proportion of both Copia and Gypsy superfamilies. Gypsy elements were contemporaneous between species, whereas Copia elements were not. Comparative genomics revealed that a quarter of gene families were contracted or expanded, including contractions in FA biosynthesis, FA elongation, and TAG synthesis genes, and an expansion in TAG degradation genes in *M. indica*. These genomic differences underpinned a single NA biosynthesis module, a reduced VLCFA biosynthesis pathway, and more degradation mechanisms, collectively leading to major VLCFA accumulation in *M. indica*. Its specialization is further evidenced by elevated FA/VLCFA biosynthesis gene expression, selection of CREs responsive to cold stimulates and chromosome rearrangements, phylogenetic core gene contraction, and reduced VLCFA gene clusters (Fig. 6). Besides, resequencing analysis divided samples from Yunnan, Guizhou, and Guangxi populations into two clades, indicating that *M. indica* of Guizhou and Guangxi populations are likely divergent from Yunnan, retaining lower diversity and showing signs of selection and recent gene flow. Overall, this study provides a genomic foundation for the targeted domestication and breeding of *Macaranga* species.

## Materials and methods

### Material collection

The *M. indica* samples were collected during the dry season (December–January), while *M. denticulata* samples were collected during the rainy season (June–July). The individuals of *M. indica* and *M. denticulata* selected for genome sequencing naturally grew in the Xishuangbanna Tropical Botanical Garden, Menglun, Mengla, Yunnan Province, People’s Republic of China. The samples for resequencing were collected from Yunnan, Guizhou provinces and Guangxi Autonomous Region, China. DNA samples were immediately vacuum-dried using allochronic silica gel, while RNA samples were rapidly frozen and temporarily stored in liquid nitrogen.

Climatic information from the habitats of *M. indica* and *M. denticulata* were collected and compiled over 3-year periods. The geographical coordinates of global *M. indica* and *M. denticulata* populations were verified using METADATA-IUCN v6.3 (https://iucn.org/), with altitude information subsequently added using ArcGIS v10.7.

### Genome sequencing, assembly, and gene annotation

Genomic sequencing was performed using both PacBio Sequel II and Illumina X Ten. Comparison was made using the NCBI taxonomy database (NT), with 10 000 (read 1 and read 2 of 5000 each) randomly selected and filtered high-quality sequences. Based on 17 *K*-mer analysis [47], genomic sequences of *M. indica* and *M. denticulata* were assembled by NextDenovo v2.2-beta.0, then accessed and revised with initial assembly by purge_haplotigs (*M. indica*)/redundans (*M. denticulata*; ident = 0.93, ovl = 0.93), and recorrected via NextPolish with the Illumina platform. They were clustered by Juicer Tools v1.9.9 and 3D-DNA v180922, and visualized or delitescent errors-redressed by Juicer Tools. Genomic sequences of *M. indica* and *M. denticulata*, measured by Hi-C, were assigned to chromosomes. The final assembly was assessed by BUSCO v3.0.2 (database: embryophyta odb10).

Gene annotation was performed using three complementary approaches: (1) homology-based prediction via tBLASTN against *A. thaliana*, *V. fordii*, *E. lathyris*, *J. curcas*, *S. indicum*, *H. brasiliensis*, *M. esculenta* and *R. communis*, followed by Genewise refinement (E-value ≤ 1e-5); (2) ab initio prediction using AUGUSTUS (with self-trained model), Genscan and GlimmerHMM, with an overlap threshold of 0.5 and A. thaliana parameters; and (3) transcriptome-based prediction using Trinity assembly of RNA-seq data, with gene models constructed by the Trimmomatic-TopHat-Cufflinks pipeline or by PASA against the reference genome. These evidence sources were subsequently integrated by EVidence Modeler (EVM).

### Repetitive sequence analysis

Homology-based prediction was combined to identify the repeat contents in our genome. Homology-based analysis identified the known transposable elements (TEs) of *M. indica* and *M. denticulata* genomes using RepeatMasker Open v4.0.9 with the Repbase TE library. Repeat Protein Mask searches were also conducted using the TE protein database as a query library. Prediction of TE constructed a repeat library of the *M. indica* and *M. denticulata* genome using RepeatModeler (https://www.repeatmasker.org/). RepeatModeler can automatically execute two core repeat-finding programs, i.e. RECON v1.08 and RepeatScout v1.0.5, to comprehensively conduct, refine, and classify consensus models of putative interspersed repeats for the *M. indica* and *M. denticulata* genomes.

We surveyed LTR retrotransposons against the *M. indica* and *M. denticulata* genome sequences using LTR Finder v1.0.7. We also identified tandem repeats using the Tandem Repeats Finder package and the non-interspersed repeat sequences (using RepeatMasker) including low-complexity repeats, satellites, and simple repeats. Finally, we merged the lib library files of the two methods and used RepeatMaker to identify the repeat contents. LTRs were detected by LTR Finder (D: 15000; d: 1000; L: 7000; l; 100; p: 20; C; M 0.9) and LTR Harvest (default), and then we conducted retrotransposon annotation, retrotransposon classification, and retrotransposon evolution analysis by LTR Retriever.

### Evolution, sequence alignment, and gene analysis

Thirteen angiosperm species, comprising *M. indica*, *M. denticulata*, six other Euphorbiaceae plants (*M. esculenta*, *R. communis*, *V. fordii*, *H. brasiliensis*, *E. lathyris*, and *J. curcas*), three NA-resource plants (*M. oleifera*, *Acer truncatum*, and *Xanthoceras sorbifolium*), the model plants *A. thaliana*, and *Amborella trichopoda*, the species sister to the remaining angiosperms, were used for evolutionary analysis by comparing the single-copy orthologs, multiple-copy orthologs, unique paralogs, other paralogs, and unclustered genes. Genomic sequences of *M. indica* and *M. denticulata* were assembled by NextDenovo and BUSCO, accessed and revised by purge_haplotigs and the Illumina platform based on 17 *K*-mer analysis, respectively. The eventual percentages of complete BUSCOs were measured at 96.30% (*M. indica*) and 97.40% (*M. denticulata*).

Upon downloaded genomes of 11 species and *M. indica* and *M. denticulata*, OrthoFinder v2.5.2 (default sets) was used to perform a similarity search for whole amino acid sequences, identifying gene families with the longest transcripts of each gene. The common single-copy orthologous genes were exacted to perform multiple sequence alignment by MUSCLE online, then RAxML v8.2.12 (−m PROTGAMMAAUTO) was used to construct a phylogenetic tree by maximum likelihood. Using TimeTree, differentiation time correction points (e.g. *Elopichthys bambusa* versus *Danio rerio*: 81.8–51.9 Mya) were rectified by MCMCtree in the PAML package by the penalty point likelihood method combined with Bayesian loose molecular clock correction. Then, gene expansions or contractions for *M. indica* and *M. denticulata* were estimated by CAFE v4.2 according to the time of differentiation and phylogenetic relationship. Using a branch-site model, positive selection was identified for each single-copy orthologous gene by CODEML in the PAML package, and *P* values <0.05 were screened by KEGG and GO analysis.

### SNP and indel analysis of *M. indica* from Yunnan, Guangxi, and Guizhou province

To investigate the genetic diversity of *M. indica* and to conserve the germplasm resources in a common garden, resequencing of 100 individuals collected from diverse geographical regions revealed genome-wide SNPs and indels. Phylogenetic trees of *M indica* populations from Yunnan and Guizhou provinces and Guangxi Zhuang Autonomous Region were reconstructed using RAxML, using maximum likelihood, based on genome-wide SNP data.

Population structure analysis was performed using SNP markers to analyse the population structure by ADMIXTURE v1.3.0 using the Bayesian mathematical model. Each individual in the material was assigned to predefined genetic clusters (*K* values) to infer hierarchical population subdivisions. The optimal *K* value was determined by minimizing cross-validation error to indicate the most biologically meaningful genetic model fitting. Treemix was performed to construct maximum-likelihood phylogenetic trees with different numbers of migration events ranging from 0 to 5, and 3 replicates for each event. Then, a web-based optM program was used to identify the optimal number of migration events [93].

Whole-genome comparisons among *M. indica*, *M. denticulate*, and the reference genome of *R. communis* were conducted using MUMmer v4.0.0 with default parameters. Adjacent intervals (≤50 bp) were merged and non-overlapping intervals specific to *M. indica* were extracted using bedtools v2.28.0. Regions shorter than 100 bp were filtered, retaining sequences as candidate PAVs (≥100 bp for *M. indica* presence variants [PVs] and reference genome absence variants [AVs]). PV candidates were aligned to *R. communis* and *M. denticulata* via BLASTN (*E*-value <10^−5^). Alignments with >50 and > 90% sequence identity were excluded to finalize the *M. indica*-specific PVs, with reciprocal filtering applied to confirm AVs. Genomic regions overlapping annotated genes were classified as PAV-associated genes. Functional enrichment analyses of these genes were subsequently performed using the KEGG and GO databases.

### RNA-seq and related analysis

RNA was extracted using the TRIzol^®^ (Invitrogen, CA, USA). RNA purity and integrity were monitored with a NanoDrop 2000 spectrophotometer (NanoDrop Technologies, Wilmington, DE, USA) and an Agilent 2100 Bioanalyzer (Agilent Technologies, CA, USA). RNA contamination was assessed using 1.5% agarose gel. Ribosomal RNA was depleted using the Ribo-Zero Magnetic Kit to enrich mRNA. Purified mRNA was fragmented into small pieces with fragment buffer at an appropriate temperature. First-strand cDNA was reflected using random hexamer-primed reverse transcription. The buffer, dNTPs (dTTP is replaced with dUTP), the DNA polymerase I, and RNase H were then added to synthesize second-strand cDNA. AMPure XP Beads (Beckman Coulter) were employed for size selection and purification, optimizing bead-to-sample ratio and ethanol concentration to maximize yield while minimizing adapter dimer formation. Following library amplification, Qubit 2.0 (Thermo Fisher Scientific) was used for quantification, and an Agilent 2100 BioAnalyzer confirmed the insert sizes. Clean reads were aligned to the genome of *M. indica* using HISAT2 v2.1.0.

FeatureCounts was employed for reads, and differential expression analysis was performed using DESeq2 v1.22.2 and edgeR v3.6.8 with an FDR (false discovery rate) threshold of <0.05, and log_2_ fold change >1 or < −1.

### WGCNA for screening genes related to nervonic acid biosynthesis

The FA composition of seed oil was determined using gas chromatography (GC) following the method described in our previous research [10]. Homogenized seed samples (~15 mg each) were methylated with 2 ml of 2% sulfuric acid in methanol solution with incubation at 85°C for 2 h. After cooling, 0.9% NaCl solution was added to precipitate proteins, and the mixture was exacted twice with 4 ml hexane. The supernatant was collected and concentrated under gentle blowing with nitrogen gas. GC analysis was performed on a PerkinElmer Clarus 680 (Singapore), equipped with a 30 m × 0.25 mm × 0.32 mm Elite-225 column (PerkinElmer, Singapore). The retention program was as follows: 150°C for 3 min, 10°C/min to 180°C for 9 min, and 5°C/min to 210°C for 8 min. The carrier gas was pure nitrogen maintained at 1.5 ml/min, and 1 ml of the diluted sample was injected. Total FA content was quantified using an absolute internal standard [94], while free acids were pre-separated by thin-layer chromatography (TLC).

WGCNA was used to identify key genes associated with NA biosynthesis during capsule maturation, involving five development stage and 12 FAs: C16:0, C18:0, C18:1, C18:2, C18:3, C20:0, C20:1, C22:0, C22:1, C22:2, C24:0, and C24:1. The co-expression network was constructed by r-wgcna v1.71 of R Studio v4.3.1 with parameters networkType = ‘unsigned,’ corType = ‘pearson’. Gene co-expression networks of *M. indica* and *M. denticulata* were constructed to make use of interaction patterns among genes. SampleTree merged the isolated units to cluster modules of related expression genes, and the pick SoftThreshold function (power) was used to estimate and select the appropriate gene co-expression network or model via scale independence and mean connectivity. FAEs and associated genes with a correlation index of >0.1 were selected as exhibiting a co-expression network, then clustered by GO enrichment and function decided by KEGG enrichment. In addition, core FAEs matching NA accumulation were circled, and top 10 related genes of each FAE of the correlation net were visualized by Cytoscape v3.10.3.

### Analysis of fatty acid biosynthesis-related genes

Package jcvi. Compara. synteny was applied to the collinearity analysis of *M. indica*, *M. denticulata*, and *R. communis*. On this basis, we determined the end locations of each fraction of chromosomes, and found related genes by the coordinates of the fracture site. Screened sequences were re-affirmed by IGV v2.17.0 by chromosome location and related references, and the promoter region.

*KCS*s of *M. indica* and *M. denticulata* were aligned, distances were computed, and a phylogenetic tree was constructed by MEGA v11.0.13. Motif identification (set: 10) and SeqLogos were analysed by MEME Suite v5.5.5. Mutant sites were distinguished by Bioedit via homologous gene alignment after revision by SnapGene Viewer v6.0.7 and IGV v2.17.0. All the above modules were coalesced by Evolview v2.0. The promoter regions in the 2000 bp before initiation codons were captured by IGV v2.17.0 and CREs were ferreted by PlantCARE. CREs were completely enumerated and spliced by TBtools-II while digital data were displayed by GraphPad Prism v9.0.

From the Identical Protein Groups of NCBI, a total of 6998 KCSs or suspected KCSs (ELO, FAE, and FAE motif-containing proteins, etc.) were aligned using MAFFT v7.0 and a phylogenetic tree was constructed online and after manually filtering off sequences of <200 bp. For the analysis of DGAT, a total of 2017 DGATs or related genes were as mentioned above. The phylogenetic tree was processed in iTOL v6 and aggregated by Adobe Illustrator 2023 to display enrichment of homologous KCSs.

Pathway of FA biosynthesis (Fig. 2B) was constructed by referring to four KEGG PATHWAY: map00061, map00062, map01040, and map00561, and manually visualized by drawio v24.6.1. The mosaics of the heat map for gene expression were colored by GraphPad Prism v9.0 and graphic designed using Adobe Photoshop 2023.

### Three-dimensional structure construction and motif analysis

#### KCSs

3D structures of KCSs were constructed by SWISS-MODEL and conjointly analysed with Pfam data; two motifs (FAE1/Type III polyketide synthase-like protein and β-ketoacyl-[acyl-carrier-protein] synthase III, C-terminal) were analysed by InterProScan. Simultaneously, CHS-like active sites, malonyl-CoA binding sites, dimer interfaces and product binding sites were extracted from InterProScan data, which assisted in motif identification, differential sequence alignment and dimensional metrology by Pymol v2.5.

#### PDATs

Modeling methods of five PDATs (Mind377G000001, Mind05G000449, Mind05G003522, Mden12G000319, Mden03G000445) were analogous to those of KCSs. Protein BLAST was executed on NCBI and ClustalW multiple alignment was used for permutating conserved regions, of which cKAJ6963324.1 [95] anatomically approximated to Mind05G000449, and Mind05G000449 was segmented into two parts: truncated 1 (1–510 of Mind05G000449) and truncated 2 with an additional M (methionine) at the 5-terminal position for recognition on the website. The seven PDATs were analysed by InterProScan to check whether active sites exist or were covered.

#### DGAT1

Human diacylglycerol *O*-acyltransferase-1 was selected from KEGG of the phospholipid pathway and was the template of 3D analysis for nine DGAT1s: Mind07G000461, Mind03G000016, Mind10G000009, Mind10G000042, Mden07G000505, Mden12G000061, Mden12G000526, Mden04G000010, and Mden10G000013. DeepTMHMM v1.0 was used to identify TM structure embracing binding sites. The 3D structures were constructed using SWISS-MODEL. Sequence alignment was performed by BioEdit v7.0.5.3 for positioning TM sequences and acyl-CoA binding sites. Protein–protein interactions were simulated on the ZDOCK server: heterologous proteins by ZDOCK v3.0.2 and homologous proteins (select symmetry = 2) by M-ZDOCK. Among 10 results from ZDOCK, we chose the most appropriate models for the following analysis on the basis of previous research [68, 69].

### Yeast one-hybrid array experiment

For expression in yeast, >1000-bp upstream promoters of *MiKCS11c*, *MiKCS11d*, *MdPPT1*, and *MiPPT1* were inserted into the pGBKT7 vector, and the CDSs of MiWRI1, MiDREB1, and MiDREB3 were inserted into pGADT7 vector. The primers are listed in Supplementary Table S47. Yeast strain Y187 was transformed with the pGBKT7 expression clone for complementation or with the unrecombined destination vector pGADT7 as a control. Transformants were selected on plates containing synthetic defined (SD) medium with the −Leu−Trp dropout supplement (Coolaber, China) for 48 h at 28°C. For each strain, an individual colony was used to inoculate 4 ml of liquid SD/−Leu−Trp medium and shaken at 200 rpm for 48 h at 28°C. Finally, yeast cells (3 μl of solution) were grown on SD/−His−Leu−Trp medium (Coolaber, China) for 48–72 h at 28°C to allow yeast growth.

### Figure visualization and statistics treatment

Visual tools used in this study were Pymol v2.5, draw.io v24.6.1, IGV v2.17.0 and Adobe Photoshop 2023. All numerical transformations, including FPKM of log_10_ fold change, log_2_ fold change, vector graph coordinates, and location transformation, were performed by Microsoft Excel 2021. Vectorgraphs were transformed by Adobe Illustrator 2023, and tiffs (Tag Image File Format) were compiled by Adobe Photoshop 2023. Chemical constructions were visualized and revised by KingDraw v3.0.0 (http://www.kingdraw.cn/).

## Supplementary Material

Web_Material_uhag114

## Data Availability

All data supporting the findings in the article and its supplementary information are available from the corresponding author upon reasonable request. Source data are provided with this paper. This whole genome assemblies of *M. indica* and *M. denticulata* have been deposited at DDBJ/ENA/GenBank under the accession JBLRME000000000 and JBKZVY000000000. The version described in this paper is version JBLRME010000000 and JBKZVY010000000.
