# Supplementary figures and images for: The chromosome-level genomes of two Macaranga species provide insights into the molecular mechanism of nervonic acid accumulation
Source: Hortic Res. 2026 Apr 2;13(8):uhag114. doi: 10.1093/hr/uhag114 (PMC13394177; doi:10.1093/hr/uhag114)

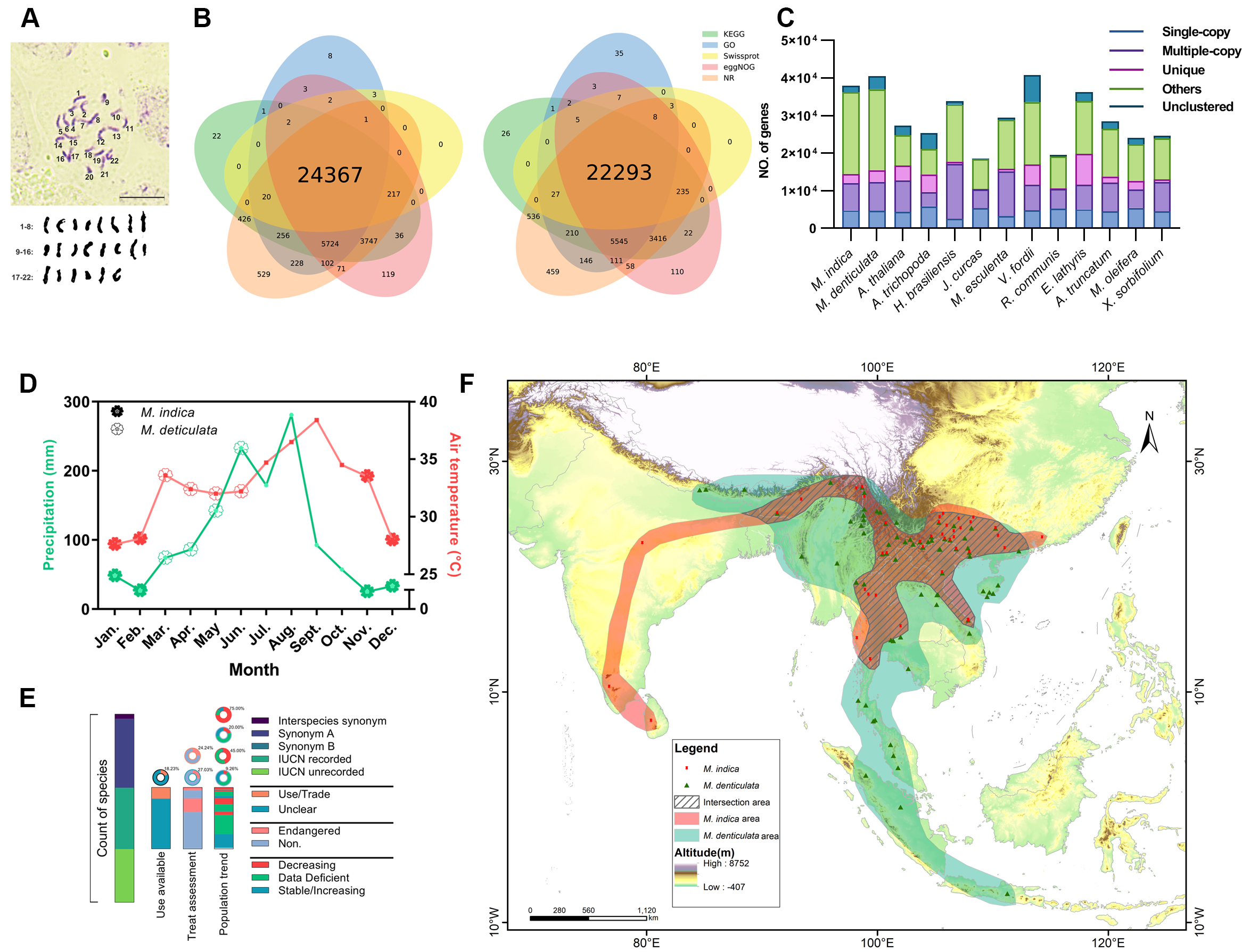

Supplement: Web_Material_uhag114 [file web_material_uhag114.zip › Figure S1-0223.tif]

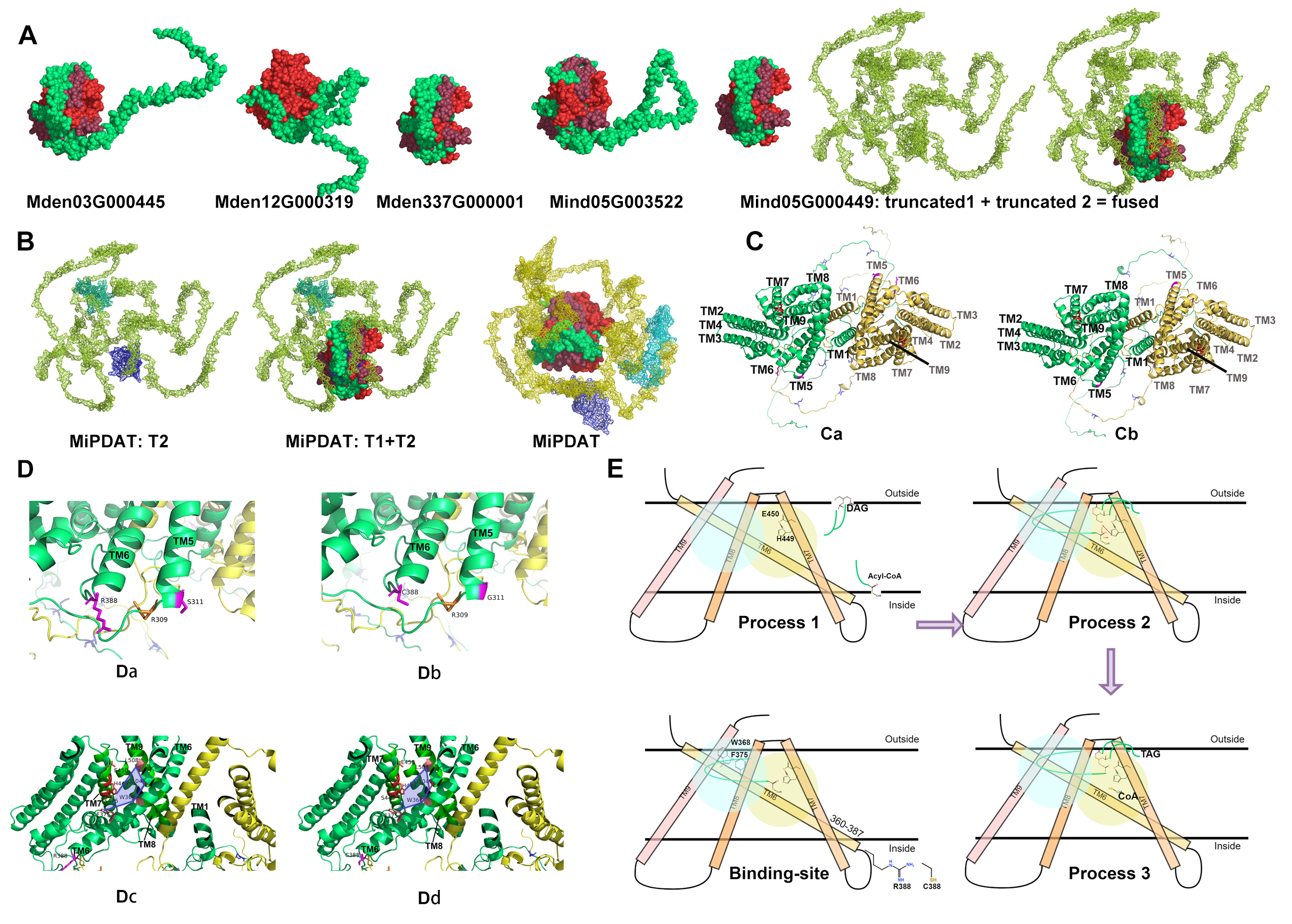

Supplement: Web_Material_uhag114 [file web_material_uhag114.zip › Figure S10-0223.tif]

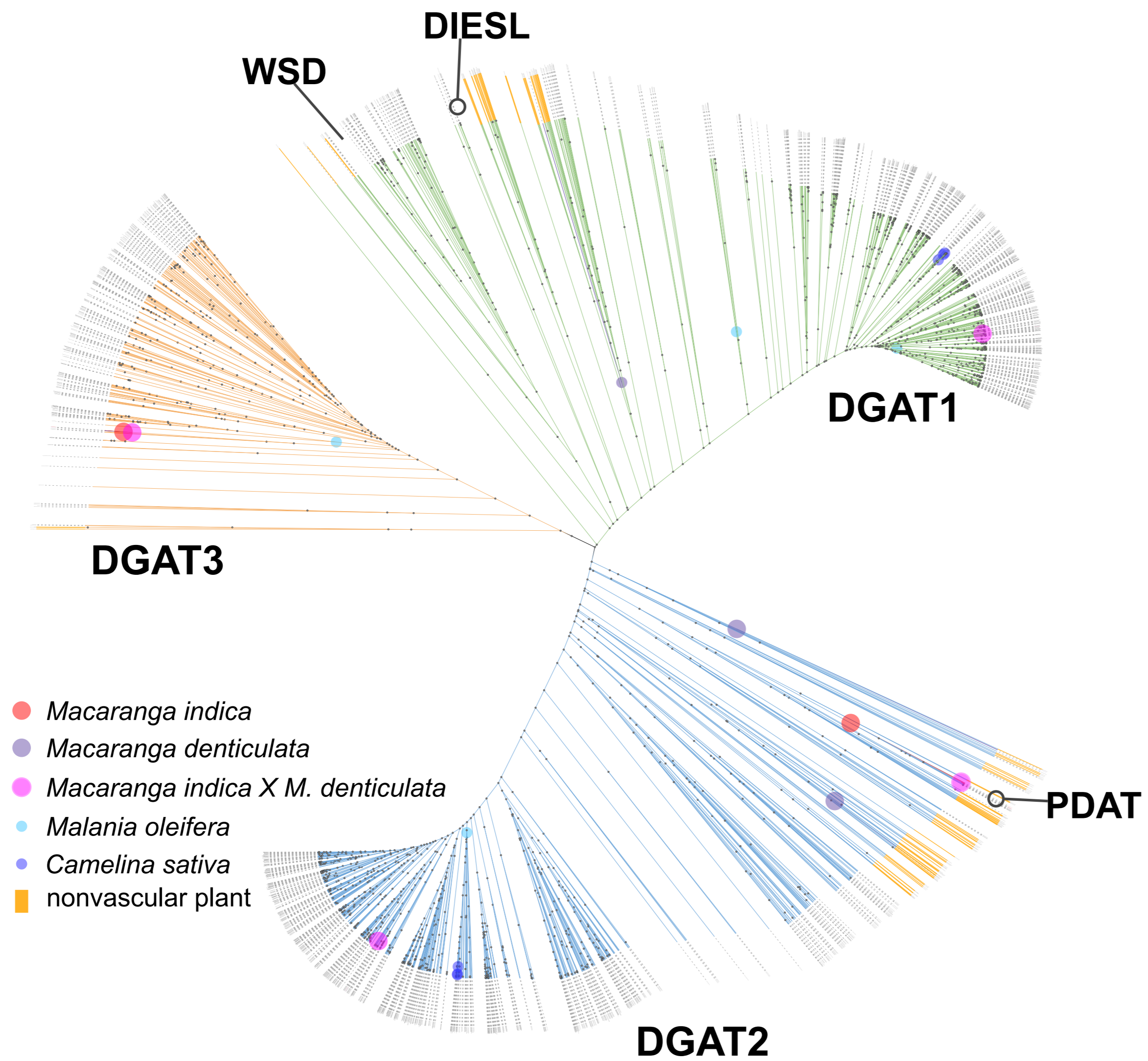

Supplement: Web_Material_uhag114 [file web_material_uhag114.zip › Figure S11-0223.pdf]

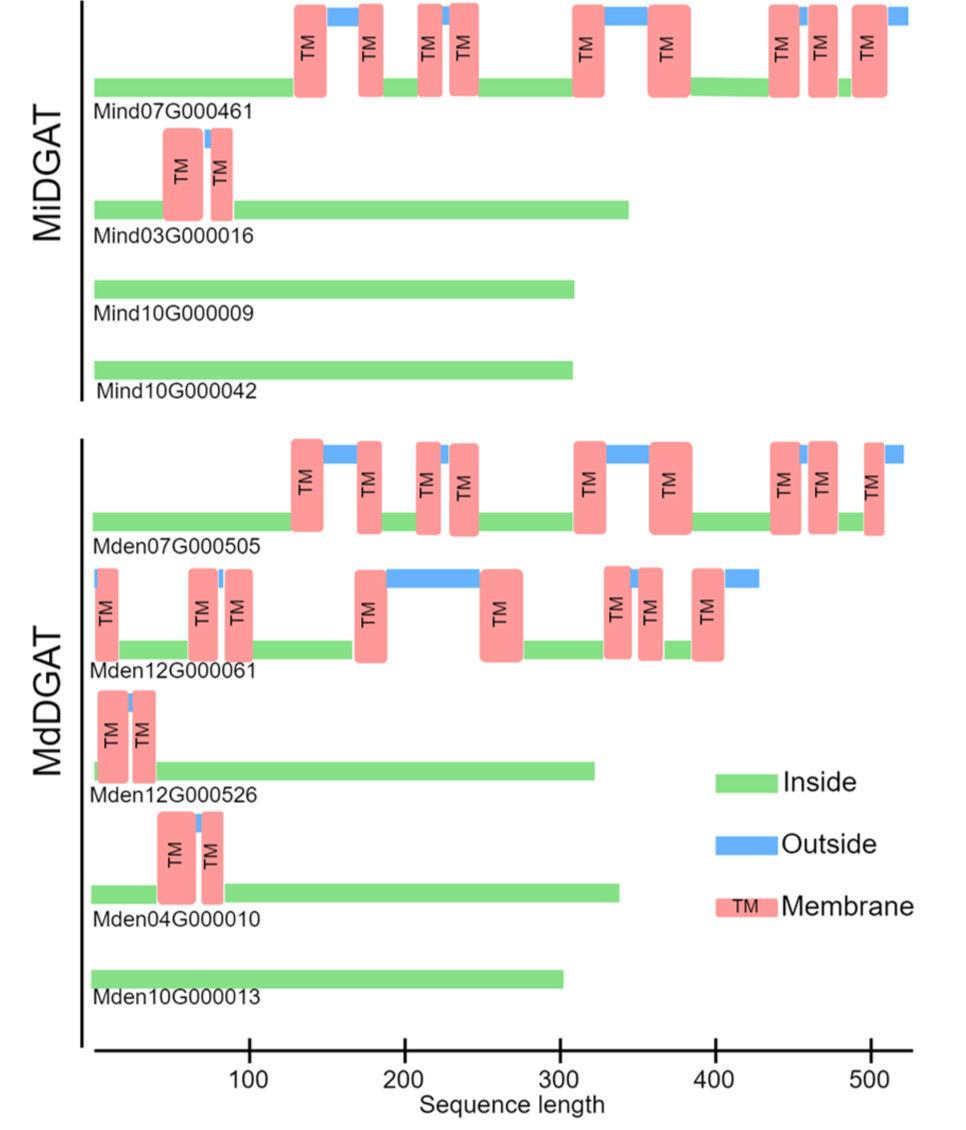

Supplement: Web_Material_uhag114 [file web_material_uhag114.zip › Figure S12-0223.tif]

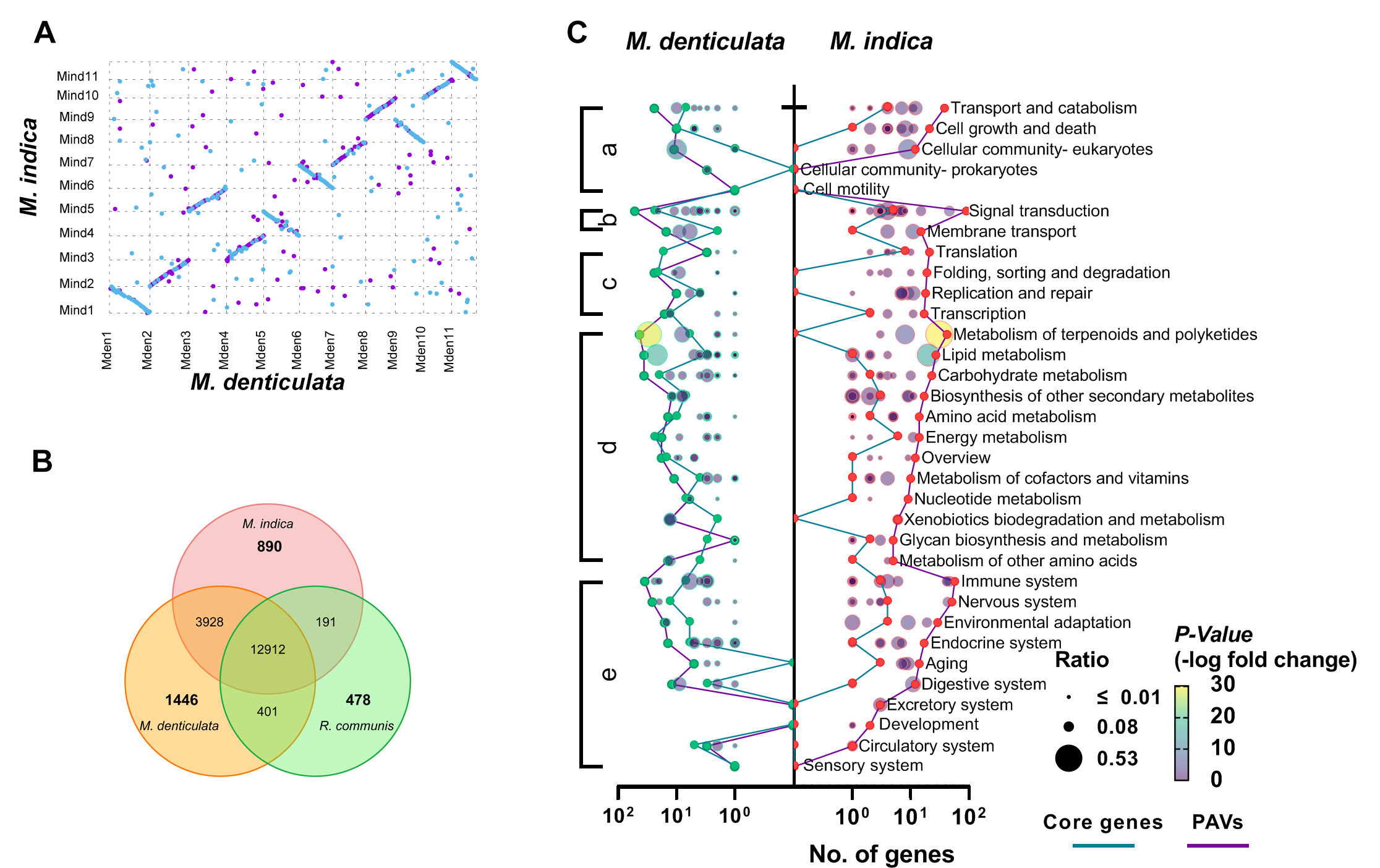

Supplement: Web_Material_uhag114 [file web_material_uhag114.zip › Figure S13-0223.tif]

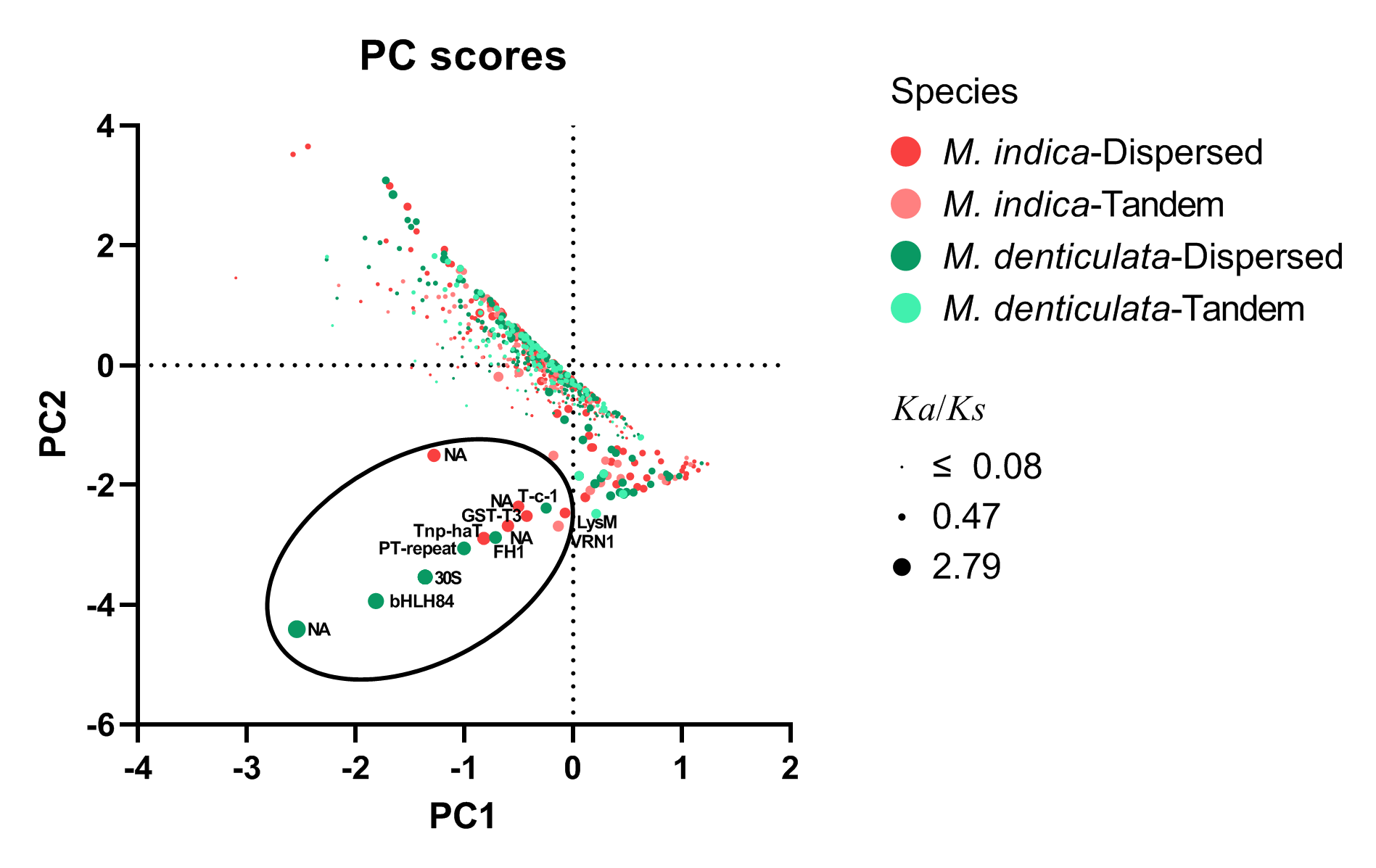

Supplement: Web_Material_uhag114 [file web_material_uhag114.zip › Figure S14-0223.tif]

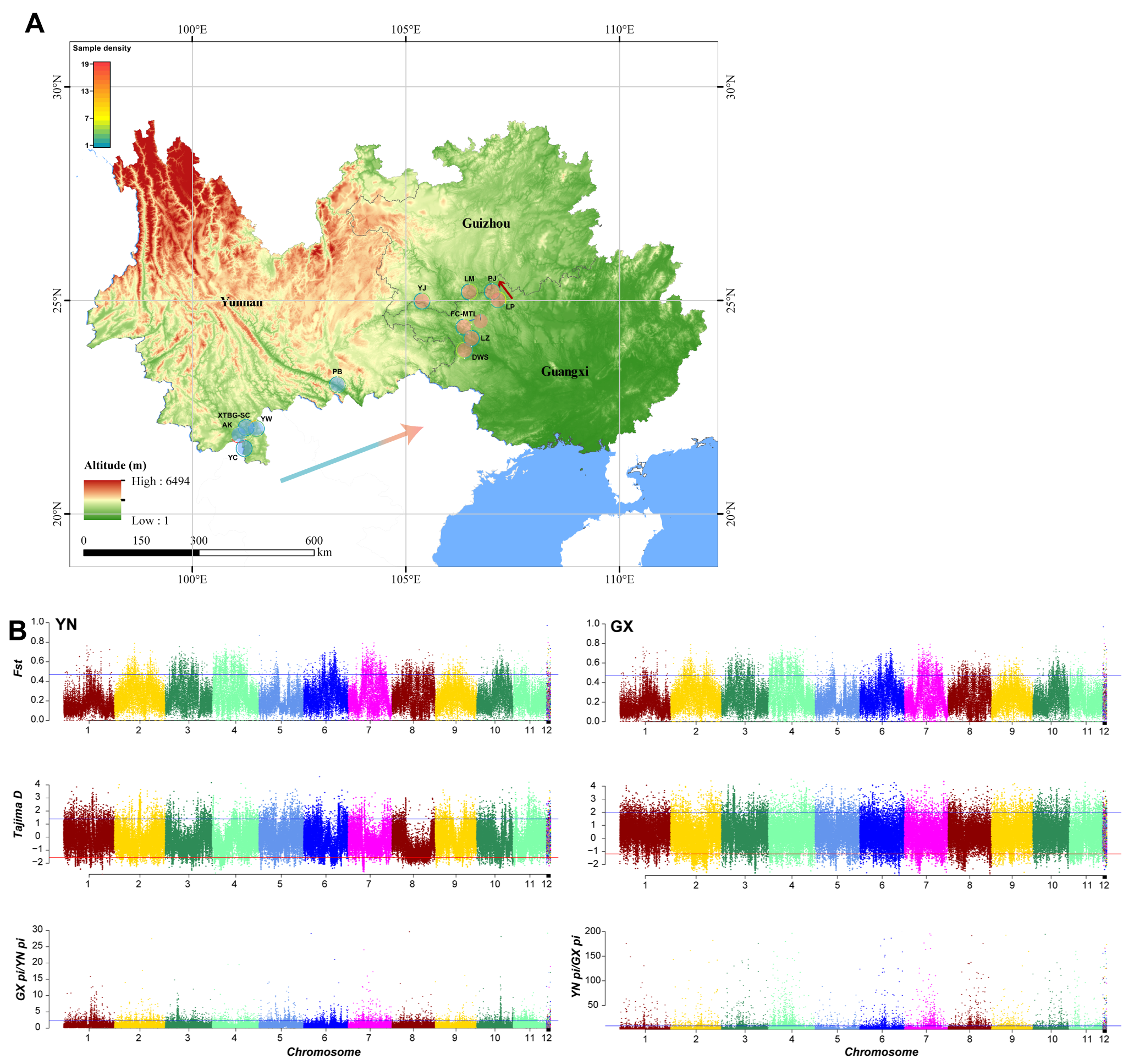

Supplement: Web_Material_uhag114 [file web_material_uhag114.zip › Figure S15-0223.tif]

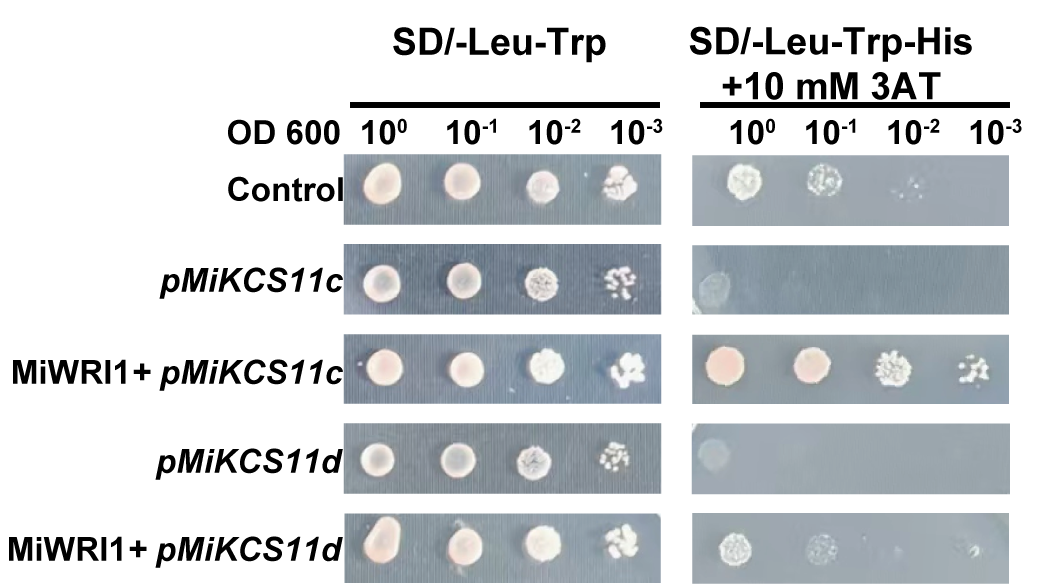

Supplement: Web_Material_uhag114 [file web_material_uhag114.zip › Figure S16-0223.tif]

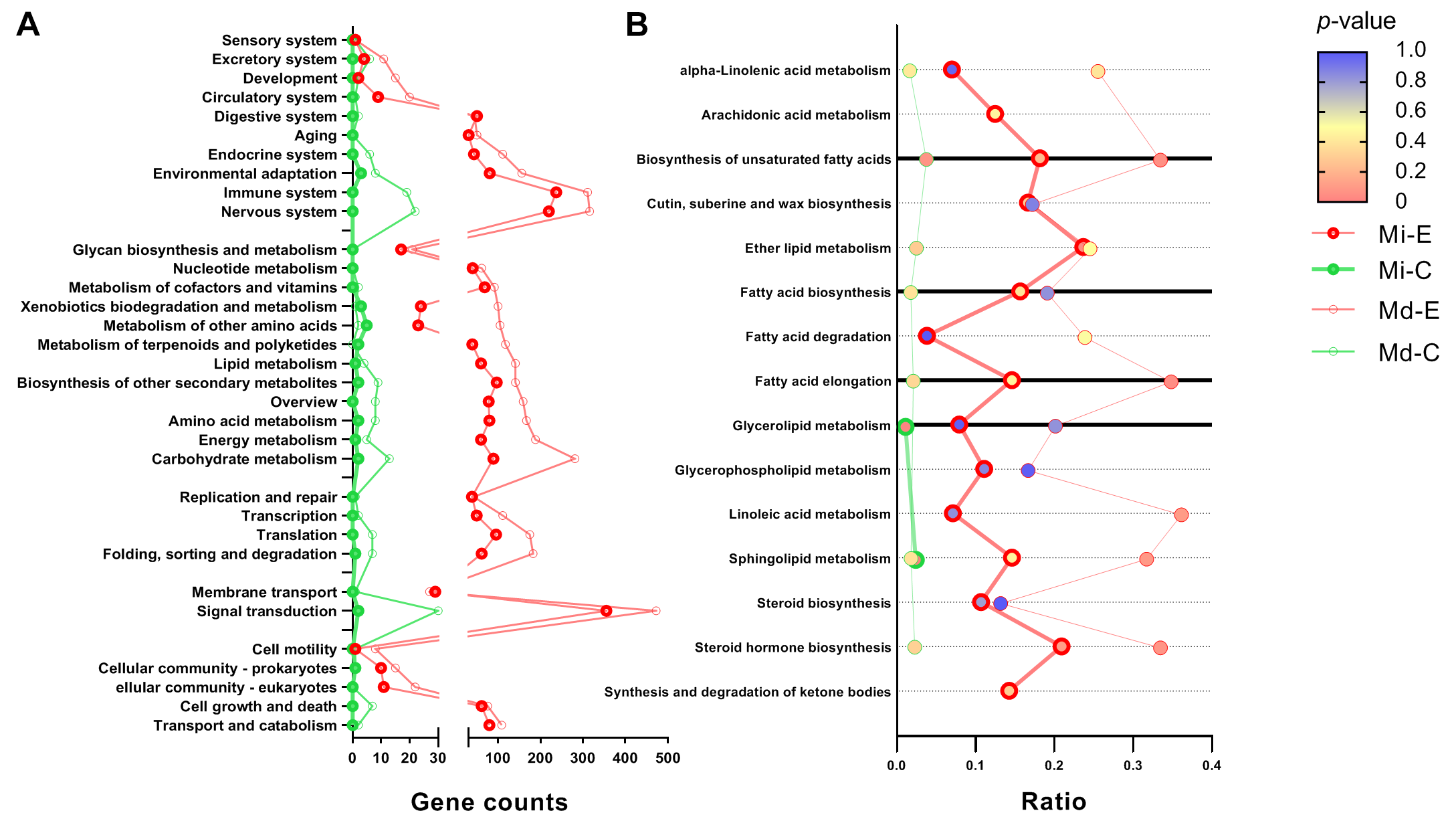

Supplement: Web_Material_uhag114 [file web_material_uhag114.zip › Figure S2-0223.tif]

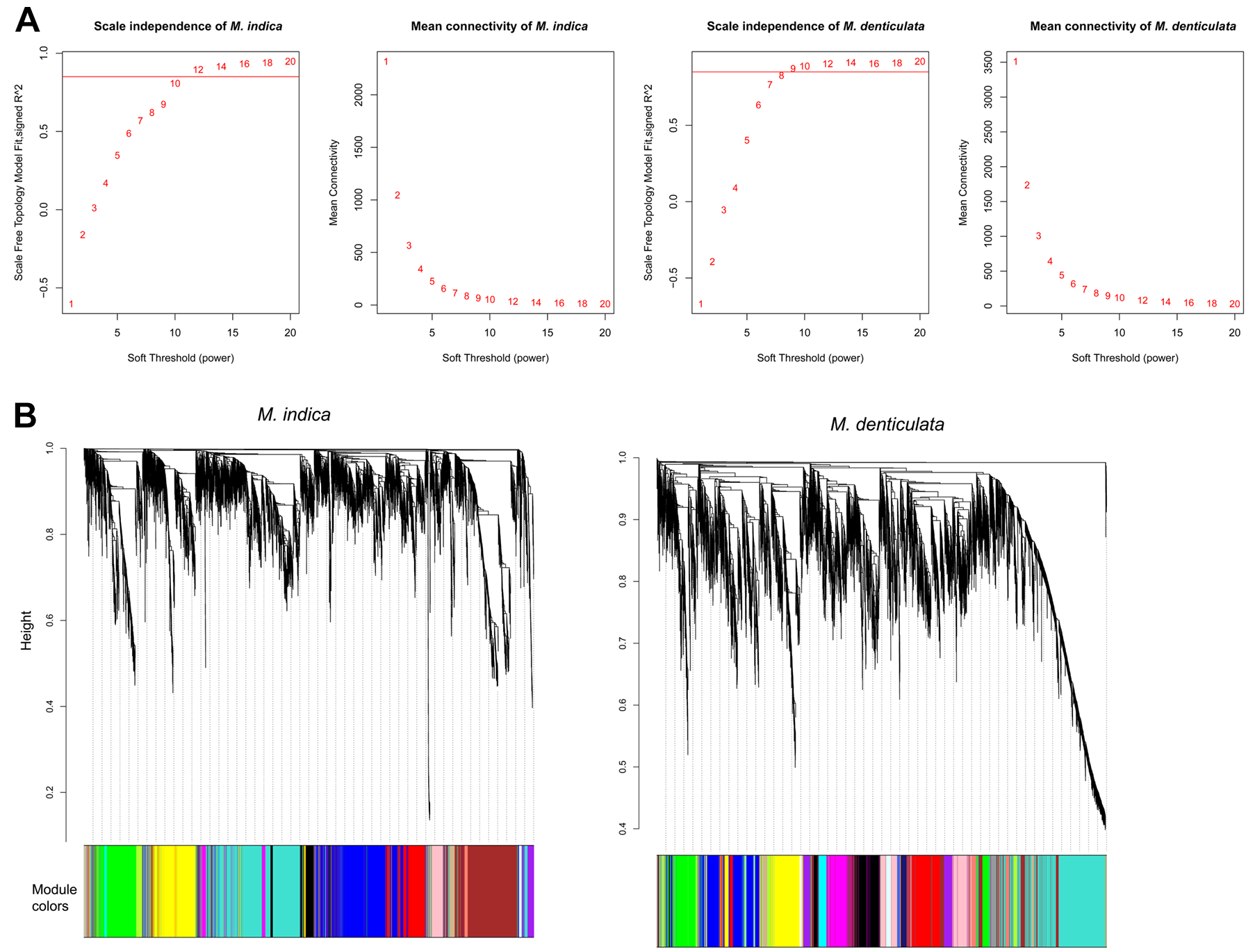

Supplement: Web_Material_uhag114 [file web_material_uhag114.zip › Figure S3-0223.tif]

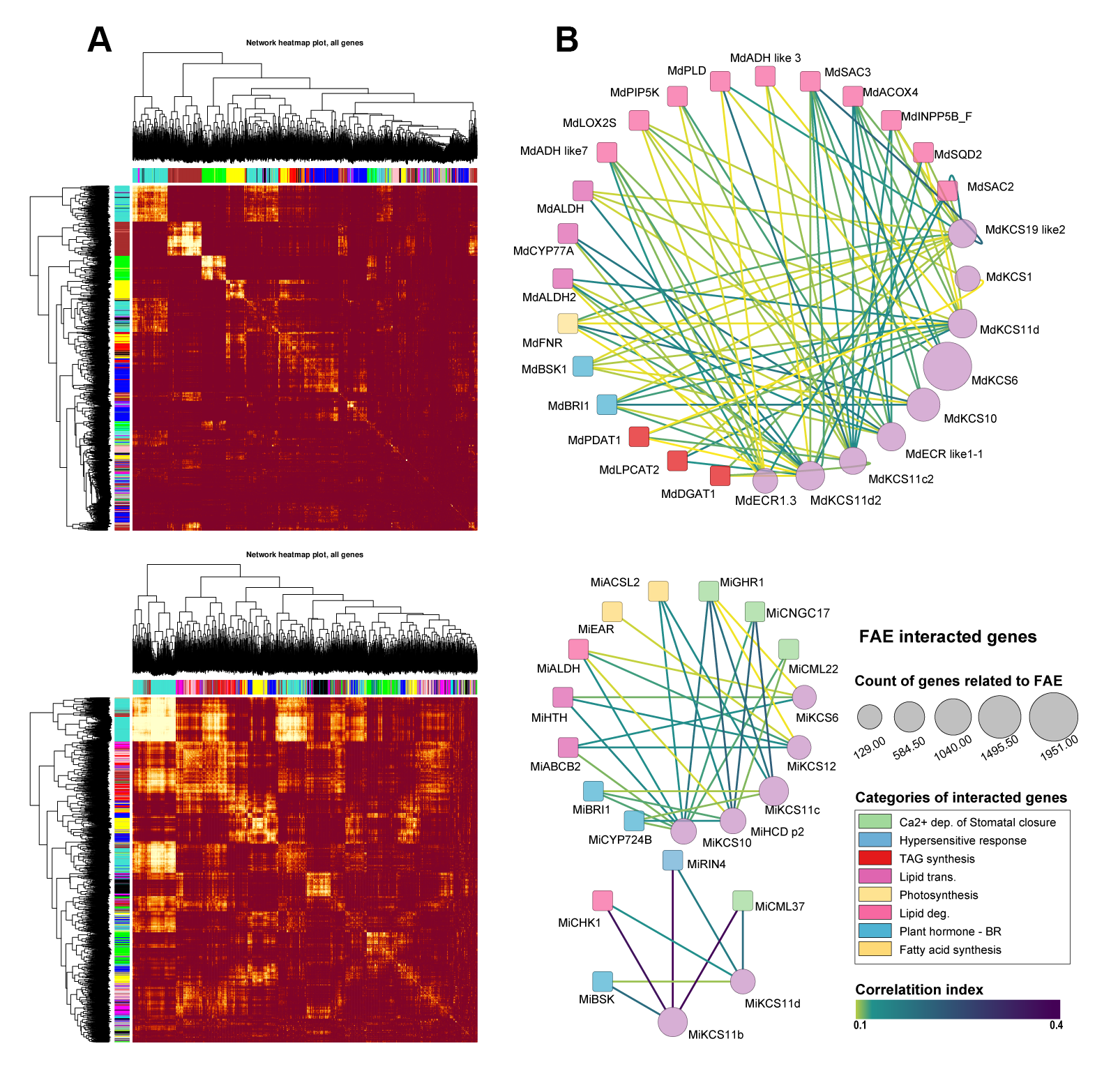

Supplement: Web_Material_uhag114 [file web_material_uhag114.zip › Figure S4-0223.tif]

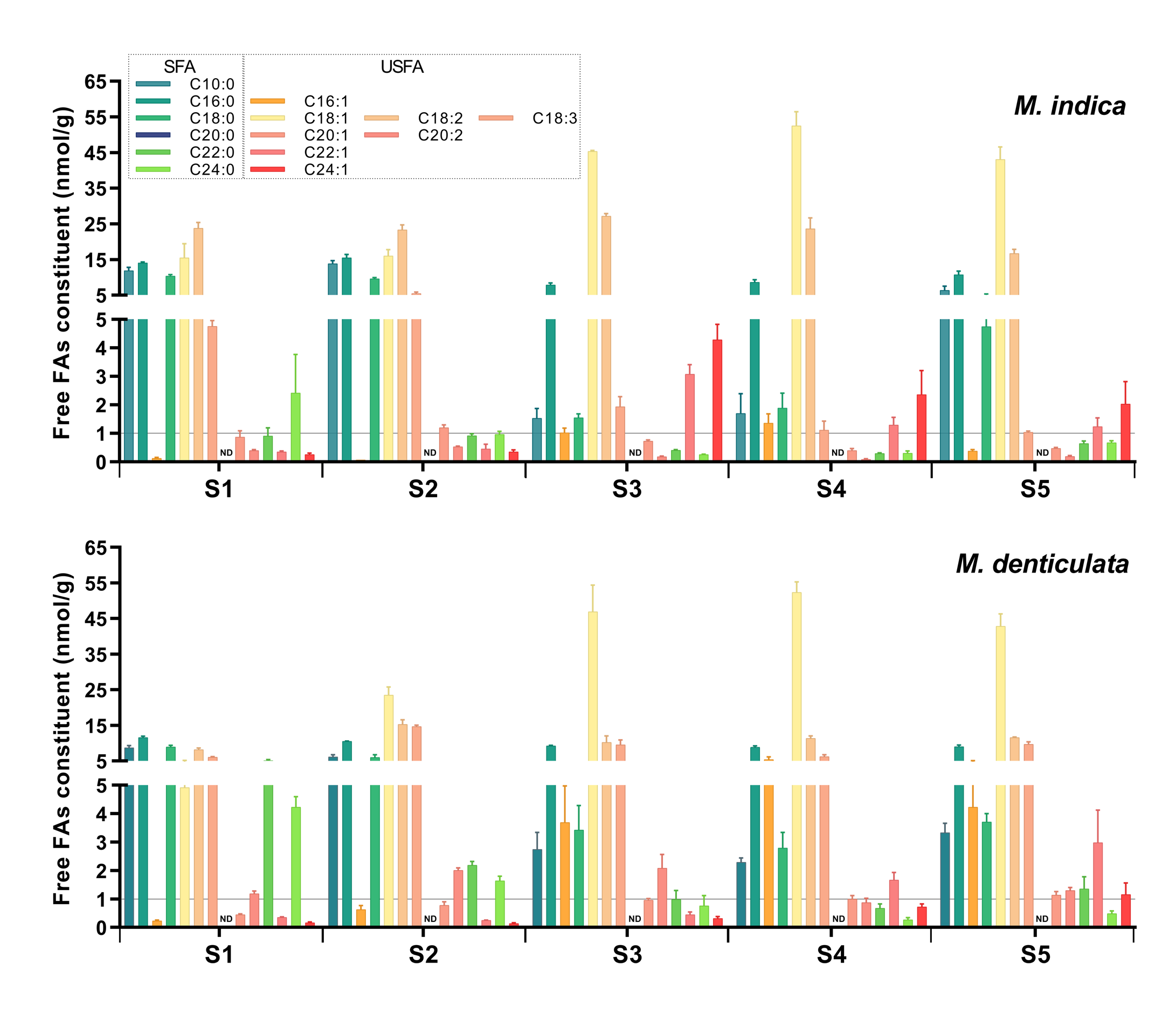

Supplement: Web_Material_uhag114 [file web_material_uhag114.zip › Figure S5-0223.tif]

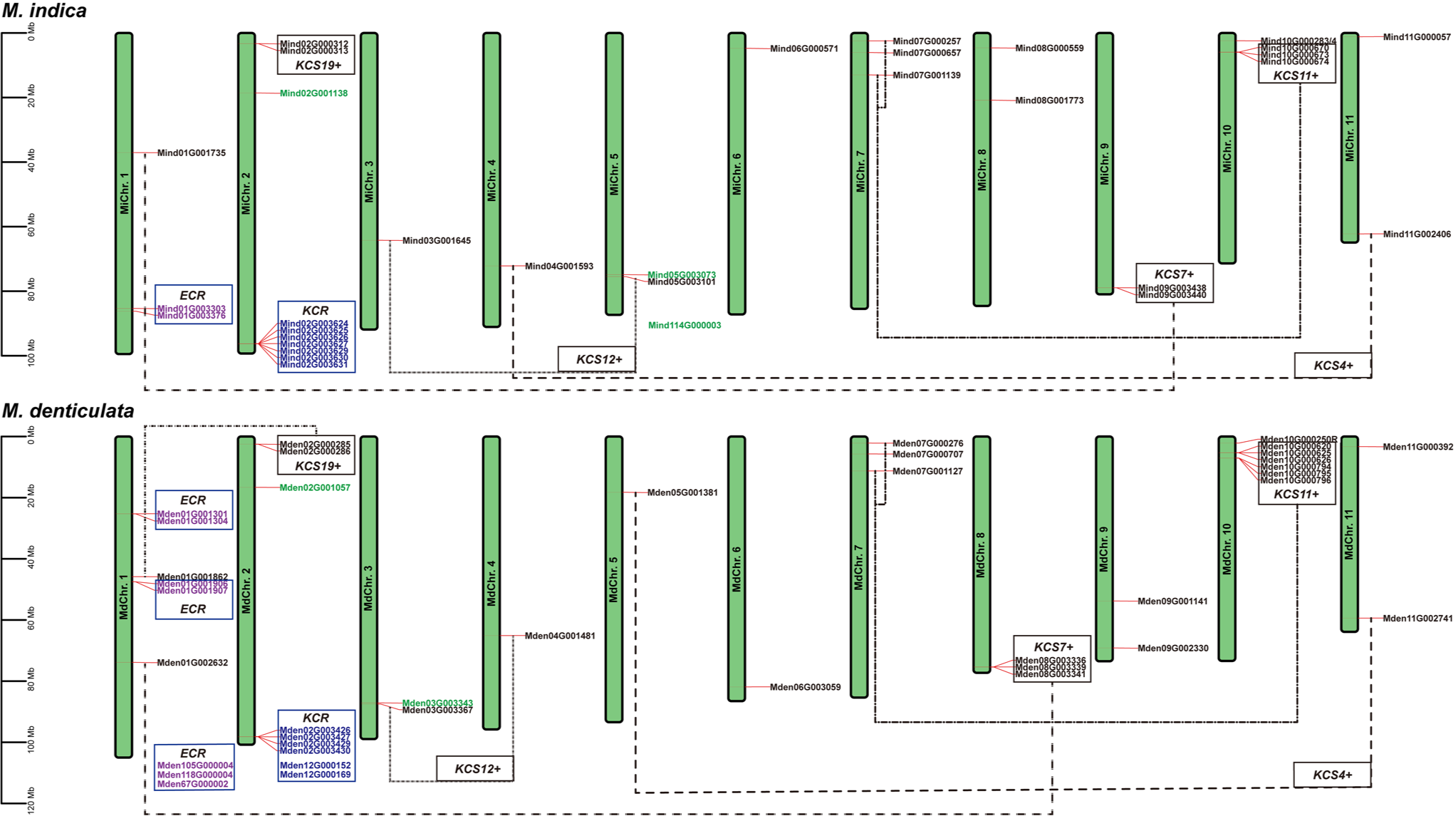

Supplement: Web_Material_uhag114 [file web_material_uhag114.zip › Figure S6-0223.tif]

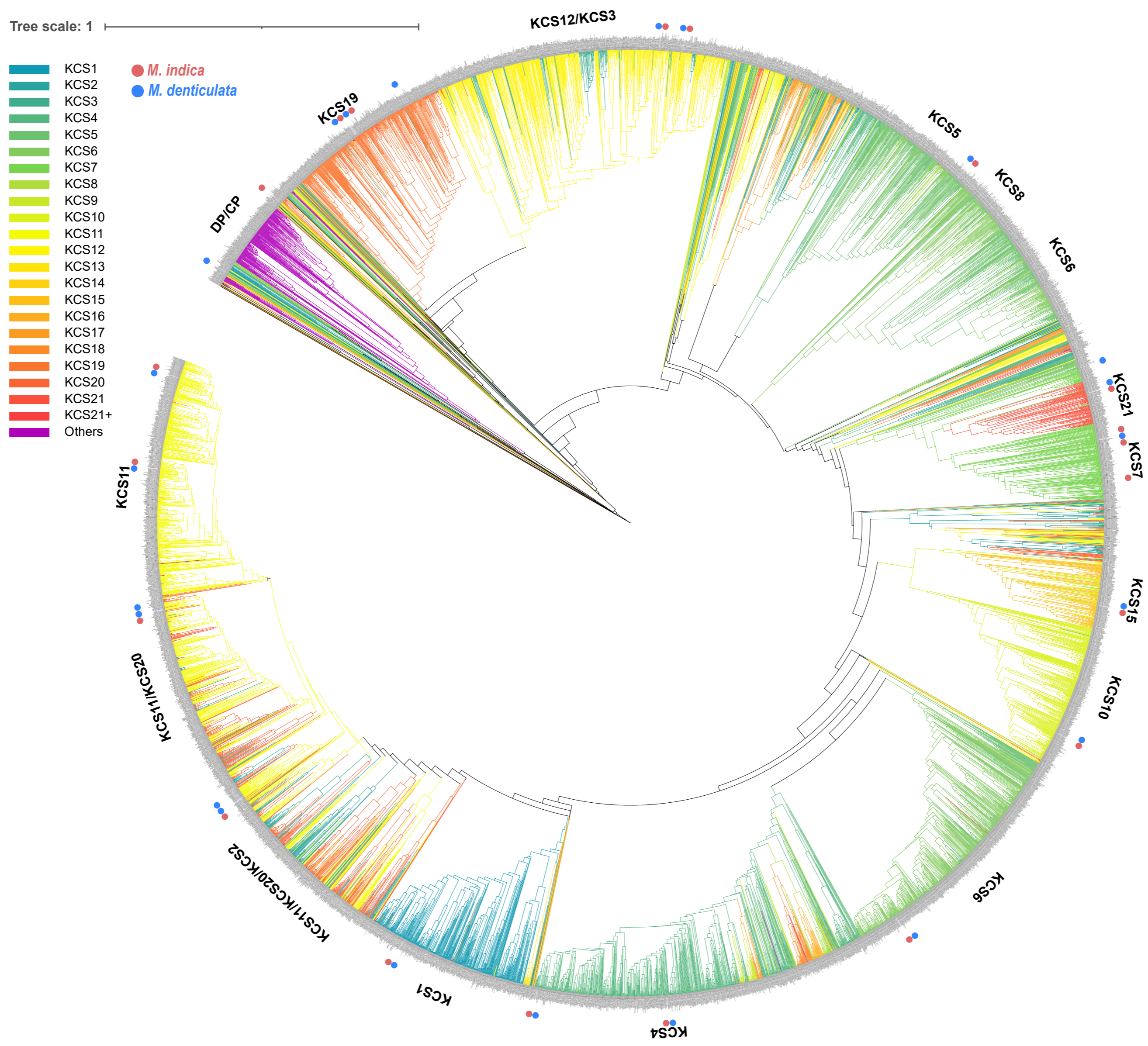

Supplement: Web_Material_uhag114 [file web_material_uhag114.zip › Figure S7-0223.pdf]

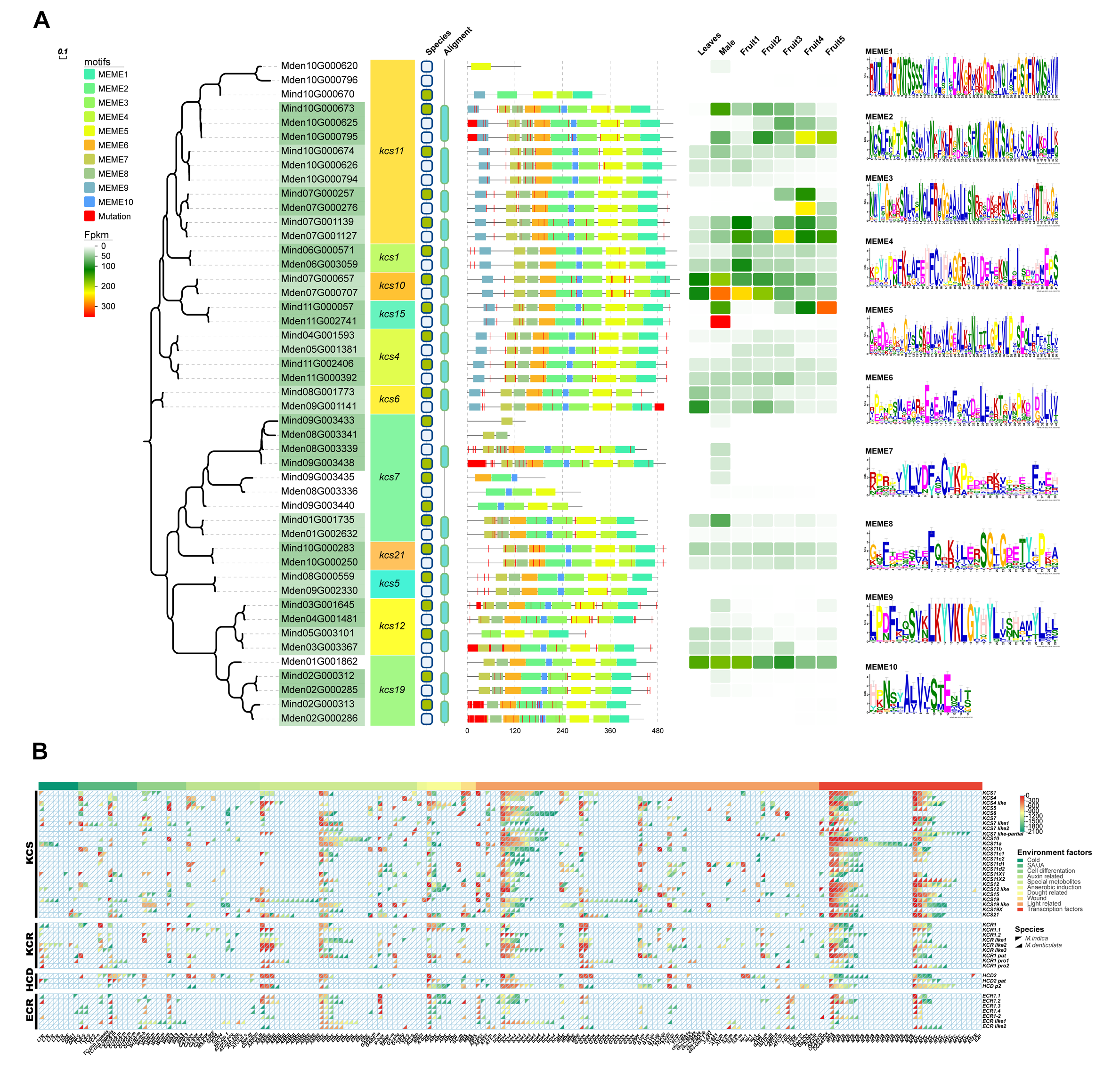

Supplement: Web_Material_uhag114 [file web_material_uhag114.zip › Figure S8-0223.tif]

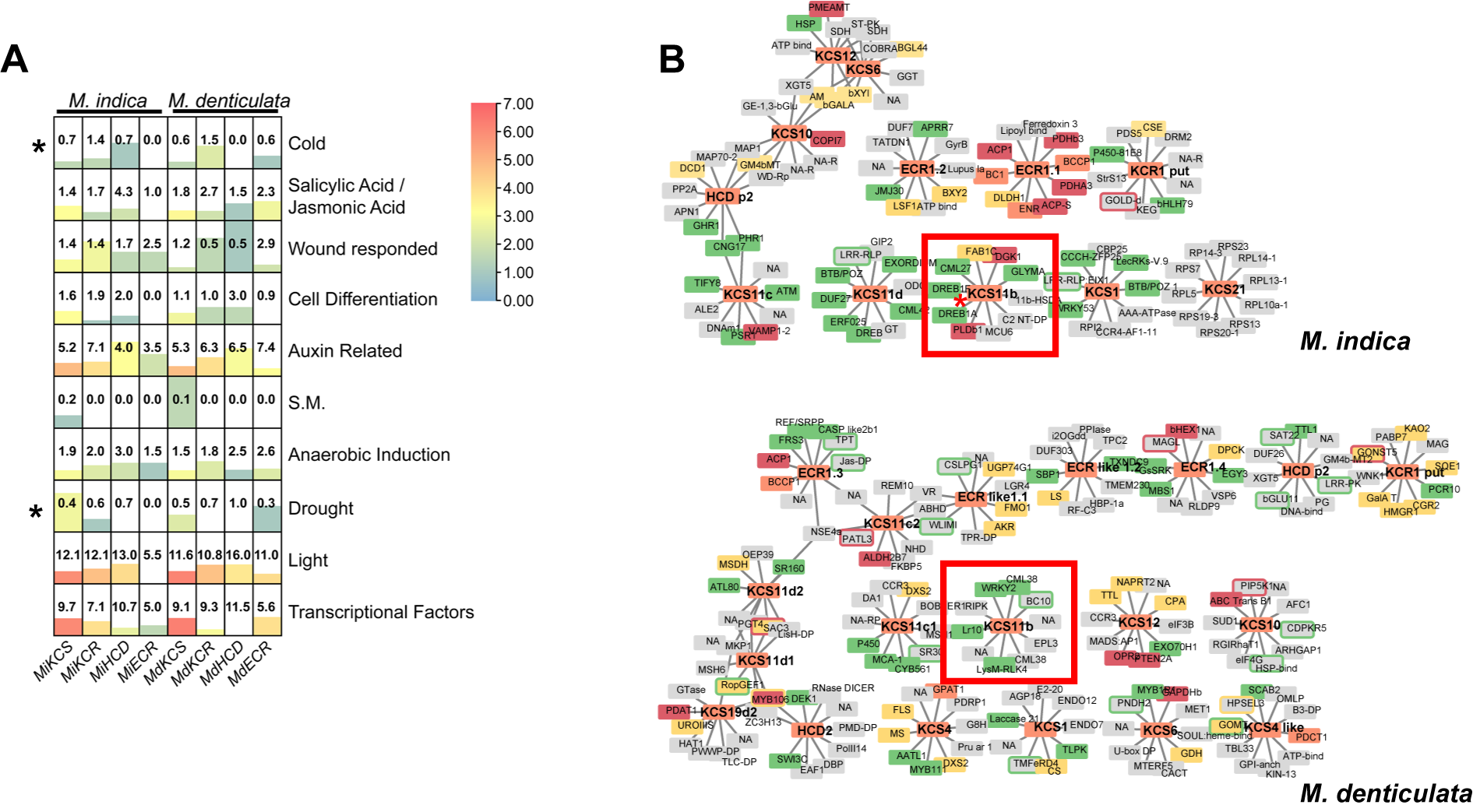

Supplement: Web_Material_uhag114 [file web_material_uhag114.zip › Figure S9-0223.tif]
